# Crosstalk among Reactive Oxygen Species, Autophagy and Metabolism in Myocardial Ischemia and Reperfusion Stages

**DOI:** 10.14336/AD.2023.0823-4

**Published:** 2024-05-07

**Authors:** Yajie Peng, Yachuan Tao, Lingxu Liu, Ji Zhang, Bo Wei

**Affiliations:** ^1^Key Laboratory of Advanced Pharmaceutical Technology, Ministry of Education of China; School of Pharmaceutical Sciences, Zhengzhou University, Zhengzhou, Henan, China.; ^2^The First Affiliated Hospital of Zhengzhou University, Department of Pharmacy, Zhengzhou, Henan, China.; ^3^Department of Pharmacology, School of Pharmaceutical Sciences, Fudan University, Shanghai, China

**Keywords:** myocardial ischemia, myocardial ischemia/reperfusion, ros, autophagy, cardiac metabolism

## Abstract

Myocardial ischemia is the most common cardiovascular disease. Reperfusion, an important myocardial ischemia tool, causes unexpected and irreversible damage to cardiomyocytes, resulting in myocardial ischemia/reperfusion (MI/R) injury. Upon stress, especially oxidative stress induced by reactive oxygen species (ROS), autophagy, which degrades the intracellular energy storage to produce metabolites that are recycled into metabolic pathways to buffer metabolic stress, is initiated during myocardial ischemia and MI/R injury. Excellent cardioprotective effects of autophagy regulators against MI and MI/R have been reported. Reversing disordered cardiac metabolism induced by ROS also exhibits cardioprotective action in patients with myocardial ischemia. Herein, we review current knowledge on the crosstalk between ROS, cardiac autophagy, and metabolism in myocardial ischemia and MI/R. Finally, we discuss the possible regulators of autophagy and metabolism that can be exploited to harness the therapeutic potential of cardiac metabolism and autophagy in the diagnosis and treatment of myocardial ischemia and MI/R.

## 1. Introduction

Ischemic heart disease (IHD) is the most prevalent cardiovascular disease, particularly in older patients [1]. During myocardial ischemia, the myocardium suffers from nutrient deprivation and hypoxia, eventually leading to cardiomyocyte death. Timely interventional strategies and the use of thrombolytic agents can improve systolic function and survival after left ventricular ischemia or infarction. Unfortunately, the sequelae resulting from rapid restoration after temporary blood and oxygen interruption, known as myocardial ischemia/reperfusion (MI/R) injury, have imposed a challenging dilemma on cardiologists.

Many hypotheses have been established regarding the pathomechanism of myocardial ischemia and/or reperfusion injury, among which autophagy has been identified as a promising orientation for further investigation. Excessive cardiac reactive oxygen species (ROS) produced during the ischemia and reperfusion stages can destroy intracellular proteins, lipids, and nucleic acids and are considered harmful to cells [2, 3]. Numerous studies have demonstrated complex crosstalk between ROS and autophagy, especially in myocardial ischemia and/or reperfusion [4]. The activation of some autophagy pathways can reduce myocardial damage by reducing the amount of ROS, and ROS can also reduce cardiac function by triggering autophagy [5]. The vicious cycle between autophagy signaling pathways and ROS leads to several deleterious effects that can be inhibited by modulating autophagic pathways. Notably, autophagy not only diminishes toxic substances to maintain cell homeostasis, but also recycles compounds to generate more nutrients and energy for basic cell survival, which plays a critical role in cell survival and cell death due to energy limitation [6]. However, the approaches for detecting cardiac autophagy in patients are limited. Therefore, it is necessary to explore novel strategies for evaluating autophagy in clinical settings.

Disordered cardiac metabolism is an important factor for cardiac injury during myocardial infarction (MI) or MI/R. Importantly, metabolomics is the effective approach to detect metabolism alterations in patients with ischemic heart disease [7]. Stress-induced autophagy degrades the intracellular energy storage to produce metabolites that are recycled into metabolic pathways to buffer metabolic stress. Thus, there is a close relationship between autophagy and cardiac metabolism. However, complex metabolic networks hinder further research.

Thus, systematically summarizing the crosstalk among cardiac autophagy, oxidative stress and metabolism disturtances in myocardial ischemia and/or reperfusion would highlight the concept of autophagic flux and strongly recognizing metabolism alterations as a platform for the analysis of autophagy. Importantly, it would be possible to detect the cardiac autophagy status via metabolics approach in clinic. This would enable the use of seek potent therapeutic strategies with promising modality, and precautionary approaches to reduce myocardial injury in patients suffering from myocardial ischemia or MI/R via regulating autophagy by mediating metabolomics abnormalities.

## 2. Reactive Oxygen Species (ROS) in myocardial ischemia and reperfusion stages

### 2.1 Definition and types of ROS

Reactive oxygen species (ROS) are highly reactive oxygen-containing chemical molecules broadly refere to oxygen radicals and non-radical derivatives of oxygen, including excited oxygen molecules, singlet oxygen (^1^O_2_^-^), oxygen-containing radicals such as superoxide anions (•O_2_^-^), hydroxyl radicals (OH^-^) and hydrogen peroxide (H_2_O_2_) [8].

### 2.2 Generation and sources of ROS in myocardial ischemia and reperfusion

In the initial stages of ischemia, ROS accumulates rapidly due to mitochondrial respiratory chain damage, xanthine oxidase (XO) activation and ferrous heme (Fe^2+^) oxidation in the oxymyoglobin complex. Following ischemia, Fe^2+^ is oxidized to Fe^3+^, which results in the production of O_2_. Subsequently, there is a surge in the amount of ROS generated during reperfusion, mainly from XO and neutrophils. Hypoxanthine can be converted to xanthine and O_2_^-^in the presence of XO and O_2_. After MI/R, neutrophils are recruited and activated, releasing toxic oxidative agents into the myocardium. In addition, the accumulation of succinate, an intermediate in the citric acid cycle, is responsible for the production of mitochondrial ROS during reperfusion, after which the accumulated succinate is rapidly reoxidized by succinate dehydrogenase, driving enormous ROS production via the reverse electron transport of mitochondrial complex I [9].

### 2.3 Mechanisms of ROS-induced damage in the heart

Under normal physiological conditions, ROS levels are precisely regulated by the activities of antioxidant enzymes such as superoxide dismutase, catalase, and glutathione peroxidase. When the production of ROS exceeds the buffering capacity of the endogenous antioxidant defense mechanism of the heart, oxidation stimulates ROS production, cellular and molecular abnormalities, cardiac dysfunction, and cardiac injury [9]. In addition to direct damage to cardiomyocytes, ROS can also induce extensive damage to lipids, nucleic acids and proteins, and induce the opening of the mitochondrial permeability transition pore (mPTP)[10, 11]. There is a vicious cycle between ROS and the ETC, as well as between ROS and mitochondrial fission, which promotes MI/R injury. ROS-induced mitochondrial fragmentation increases autophagy, apoptosis, and necrosis [11].

## 3. Cardiac Autophagy during myocardial ischemia and reperfusion stages

### 3.1 Definition and role of autophagy in cellular homeostasis

The accumulation of unusable by-products may threaten the normal function of the organism. For mutual homeostasis and keeping physiological functions normal, two exquisite degradation systems are inscribed in eukaryotic cells’ genes: ubiquitin proteasome pathway and autophagy lysosome pathway. The former is mainly responsible for selective degradation of intracellular short-acting proteins, while the latter evolved to degrade long-lived intracellular proteins and damaged organelles [12]. Autophagy is a highly conserved self-protection mechanism that has evolved to reduce unfolded and misfolded proteins, protect cells, and produce more nutrients, such as fatty acids and free amino acids, to promote the survival of essential cells [13]. Autophagy (from the Greek, "auto-" self, "-phagy" to eat) is a lysosome dependent bulk degradation mechanism, which targets cytoplasmic substances including cytosol, macromolecular components and organelles that are no longer needed, and is essential for the maintenance of cellular homeostasis [14, 15]. It maintains energy metabolism homeostasis under nutrient deprivation and maintains cellular quality by eliminating damaged proteins and organelles [16].

**Figure 1.**
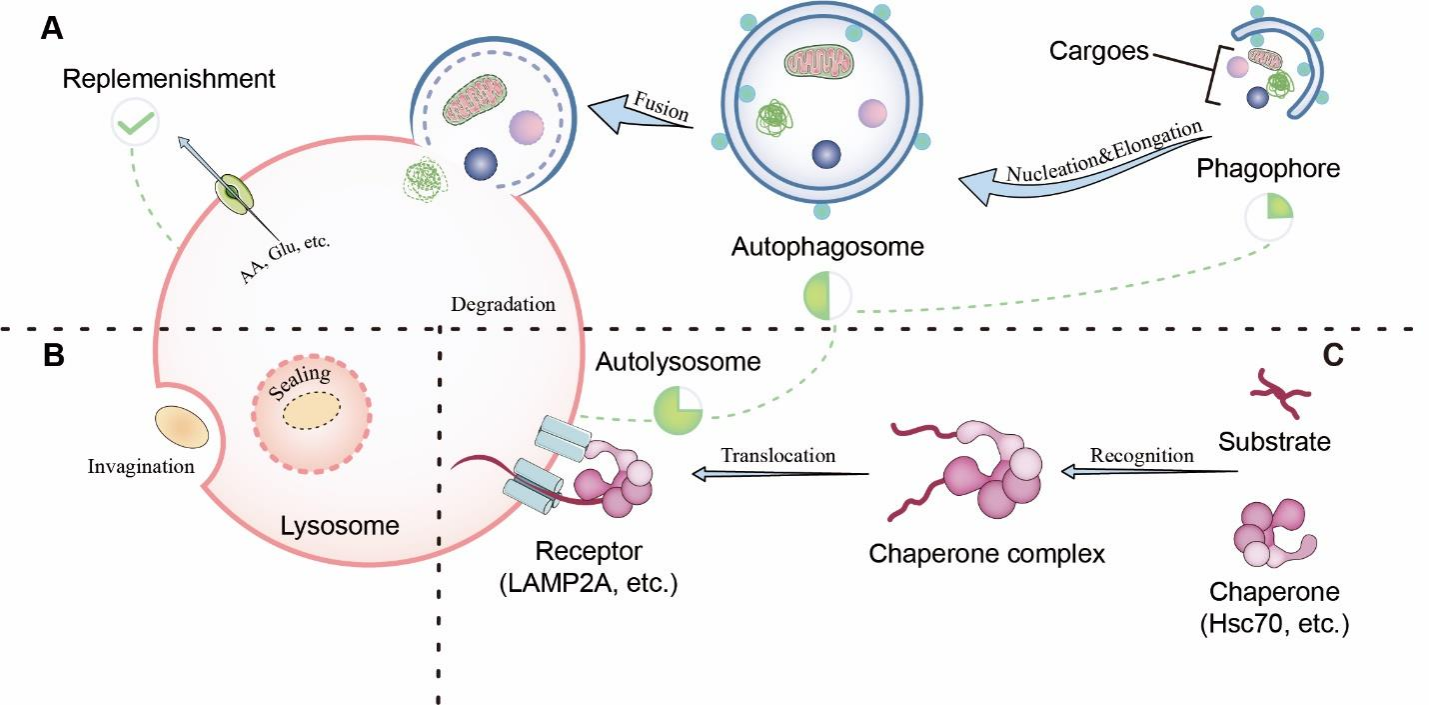
**Overview of autophagy**. Three broad types of autophagy, including macroautophagy, microautophagy, and chaperone-mediated autophagy.

Based on the unique initiation mechanism and transfer approach to lysosomes involved in mammalian biosystems, researchers have categorized autophagy into three types (Fig. 1): Macroautophagy, microautophagy, and chaperone-mediated autophagy (CMA) [17]. Macroautophagy, often referred to as autophagy, is characterized by the encapsulation of cargo in the cytoplasm into double membranes (i.e., autophagosomes), which are then delivered to lysosomes where they are further degraded and replenished by enzymatic catalysis [18]. Microautophagy is the invagination or protrusion of various organelles and proteins into lysosomes or vacuolar membranes to form vesicles for degradation [19]. For CMA, certain cytoplasmic proteins are recruited to the external surface of lysosomes in the form of substrate chaperones and no bulk engulfment is involved in process [20]. Although autophagy was initially thought to be non-selective, mitochondrial health flourishes and operates in an organelle-selective manner, with profound implications for mitochondrial health. The cellular and molecular mechanisms underlying macroautophagy have been studied extensively. Therefore, in this review, we focused on macroautophagy (hereafter referred to as autophagy).

### 3.2 Regulation of autophagy in myocardial ischemia and reperfusion

#### 3.2.1 Early neglect: does autophagy exacerbate or mitigate cardiac injury?

Cardiomyocyte defects and excessive autophagy can lead to cardiomyocyte death. As a cellular process responsible for substrate degradation, when autophagy is over-activated beyond a certain level, essential proteins or organelles are removed, resulting in cellular dysfunction and autophagic death. Mitophagy, a protective form of autophagy during MI/R injury, removes dysfunctional mitochondria and prevents inflammatory responses and excessive ROS production, thus protecting cardiomyocytes [21]. However, supraphysiological levels of autophagy may induce the excessive destruction of cellular components, leading to cell death during both macroautophagy and mitophagy [22]. Therefore, excessive autophagy exacerbates injury, which further reinforces the idea that genetic or pharmacological blockade of autophagy can improve cell survival in some cases [22]. However, impaired mitophagy can lead to the accumulation of discarded mitochondria and apoptosis, resulting in mitochondrial dysfunction and interruption of cardiac homeostasis, thereby increasing the size of MI and worsening ventricular function [23]. However, whether autophagy exacerbates or mitigates the disease pathogenesis remains controversial. More effective techniques and methods are needed to measure autophagy and mitophagy, considering different time periods and stimuli, and the complex interplay of mitochondrial dynamics and mitophagy.

However, investigations have revealed impaired autophagosome clearance in vivo and in vitro, together with decreased levels of lysosomal-associated membrane protein-2 (LAMP2) and increased levels of Beclin 1. Moreover, defective clearance was sufficient to induce cardiomyocyte injury [24]. That is, deleterious cargo is related to the severity of injury owing to insufficient clearance of autolysosomes and is probably related the increased prevalence of ROS and mitochondrial permeabilization. Intriguingly, the number of autophagosomes does not represent overall autophagy intensity: the number of autophagosomes may increase either by induction of the initial process or by accumulation due to the blockage of further consumption [25].

#### 3.2.2 Autophagic flux in myocardial ischemia and reperfusion stages

As demonstrated above, the process of autophagy consists of four dynamic stages known as autophagic flux (Fig. 1): 1) Nucleation: biogenesis of pre-autophagosomal phagosomes (PG, isolation/sequestration membranes, used interchangeably below) occurs around the organelle or protein to be degraded in response to peripheral stimuli; 2) Elongation: the isolation membrane continues to extend to form a sequestrating compartment containing the cytoplasmic components known as the autophagosome (AP); 3) Fusion: autophagosomes identify and fuse with lysosomes (LY) to form autolysosomes (AL); 4) Degradation: the contents of the sequestration are eventually degraded by various hydrolytic enzymes present in the lysosome.

Defects in autophagic flux were initially observed in inherited cardiovascular diseases, including Danon and Pompe diseases [26-28]. Recently and notably and has been considered important pathological mechanisms of MI/R injury [29]. The complete autophagic flux process involves the entire process of autophagosome formation, autolysosome formation, and content degradation. When the rate of autophagosome formation exceeds that of autophagosome degradation, autophagic flux is blocked, resulting in the accumulation of excessive autophagosomes. Autophagosomes are degraded by fusion with lysosomes to form autolysosomes, a highly regulated process modulated by protein levels and proteasomal pH [30]. The maturation of autophagosomes or defects in lysosomal function can lead to the cessation of autophagic flux. Specifically, autophagosome maturation is regulated by several factors, such as the E1 ubiquitin-activating enzyme, cathepsin D, and the small GTP-binding protein Rab7 [31, 32]. Lysosomes comprise soluble acid hydrolases, integrated membrane proteins, and membrane-associated proteins. Dysfunction of any of these components may lead to lysosomal defects, prevent the normal formation of autolysosomes, and lead to the accumulation of autophagosomes. For example, LAMP2 reduction associated with the lysosomal membrane leads to lysosomal dysfunction [32]. In MI/R, autophagosome maturation or impaired lysosome function prevents, autophagosomes and lysosomes from fusing normally, which blocks autophagic flux and further damages protein aggregation, thus destroying cell homeostasis and damaging cell functions [4]. Previous studies have shown that MI/R-induced myocardial injury can be alleviated by inhibiting excessive formation of autophagosomes and promoting their clearance of autophagosomes to increase autophagic flux [33].

#### 3.2.3 Assessment of autophagic flux

Researchers have monitored autophagy activity by observing the morphologically characteristic vacuoles that form in cells via transmission electron microscopy or by directly measuring the turnover ratio of autophagosomal proteins such as LC3 and the GABAA receptor-associated protein (GABARAP) family. There are two forms of LC3. During autophagosomes formation, LC3 is transformed from LC3-Ⅰ in the cytoplasm to the LC3-Ⅱ covalently conjugated to the membrane until it is recycled or degraded by lysosomes. Accordingly, the level of LC3-II roughly correlates with the number of autophagosomes. During the MI/R period, excessive accumulation of damaged organelles and proteins is observed, accompanied by overformation of autophagosomes, which is often manifested as increased autophagosomes observed by electron microscopy, increased LC3-II/ I ratio, and increased expressions of LC3-II and Beclin1 by Western blot assays [30]. Intriguingly, the number of autophagosomes at a specific time point does not invariably correspond to the overall autophagy intensity; the large number of autophagosomes may increase either by the induction of the initial process, or by accumulation due to the obstruction of further consumption. Limited consensus on this intracellular surveillance system has been reached, and vigorous criteria based on the integrity of autophagy are urgently needed.

Furthermore, autophagic flux is ultimately aimed at evaluating how much and how fast cytosolic constituents or mitochondria are catabolized in autolysosomes per unit of time [34]. Currently, the LC3 dual fluorescence labeling method is widely used to observe changes autophagic flux [24], the essence of this method is to quantify autophagic flux by tandem monomeric probe: red fluorescent protein (mRFP, or mCherry)-GFP tandem fluorescence labeled LC3 (mRFP-GFP-LC3, tf-LC3). This reporter emits yellow signals (emerged images of GFP and mRFP/mCherry colocalization) in the cytoplasm and autophagosomes, but only red signals are left in autolysosomes (with lower pH), because GFP is relatively susceptible to quenching and/or degradation in acidic environment than its counterpart [35]. It should be noted that this monitoring system only quantifies the flux of the reporter to acidified lumens, and practical degradation efficiency requires additional analysis, as both autophagy induction and blockade at later steps give rise to a similar accumulation of autophagosomes.

Another strategy to compare flux variation *in vivo* is to evaluate the degeneration intensity under treatment with fusion or lysosomal blockers. Multiple pharmacological agents are utilized in autophagic flux research [36] to impede lysosome-autophagosome fusion, including bafilomycin A1, chloroquine (CQ), and ammonium chloride, and to inhibit lysosomal proteases such as E64d, and leupeptin.

Furthermore, based on the peculiar maturation trait, Kaizuka et al also developed a novel single molecule-based fluorescent probe: GFP-LC3-RFP-LC3ΔG [37], which is a superior in evaluating total cellular autophagic intensity and high-throughput drugs screening. With endogenous ATG4 proteases, the probe is cleaved into equimolar amounts of GFP-LC3 (conjugated with autophagosome) and RFP-LC3ΔG. The latter moiety is recycled back to the cytosol, serving as an internal control, and autophagic activity is quantitatively monitored by the GFP/RFP signal ratio (reversely correlated). Table 1 lists the current autophagic flux monitoring assays, their quantitative indices, and their *in vivo* applicability.

**Table1:** Summary of monitoring assays of autophagic flux.

In vitro assay	Quantification	Quantitative indexes or methods	In vivo application	Ref.
Electron microscopy assay	Semi-quantification	Counting autophagosome numbers or evaluating vacuole size.	—	[30]
mRFP-GFP-LC3	Semi-quantification	Yellow fluorescent dots in merge represent autophagosomes and red represent autophagosomes under fluorescence microscopy	mRFP-GFP-LC3 transgenic mice	[35]
GFP-LC3 fluorescence microscopy assay	Semi-quantification	Number of LC3 puncta under fluorescence microscopy	GFP-LC3 transgenic mice	[24]
Immunoblotting assay of autophagosome indicators (LC3 and p62)	No	Relative expression levels of LC3 and p62 and its alterations between before- and after- treatment by lysosomal enzymes inhibitors.	—	[36]
tf-LC3 quenching assay	Semi-quantification	GFP intensity.	tf-LC3 transgenic mouses	[35]
GFP-LC3-RFP-LC3ΔG	Semi-quantification	GFP/RFP signal ratio.	GFP-LC3-RFP-LC3ΔG transgenic mouses	[37]

In summary, investigators must determine when to evaluate the levels of early or late autophagic compartments, or autophagic flux. New interpretations have indicated that autophagic flux is partly impaired rather than further maladaptively promoted, and divergence between the rate of generation and the rate of conservation into autolysosomes occurs during MI and MI/R [24, 38]. Thus, attention should be paid to garbage entering the “garbage transfer station,” which is disposed of rather than only sequestered, to aggravate cardiomyocyte damage. Pharmacologically, enhancing autophagic flux or alleviating the blockade of late autophagy, instead of neglecting the integrity of autophagy, is a promising strategy for achieving myocardial salvage in the ischemia and reperfusion stages. Therefore, detecting metabolic alterations would be an effective strategy to evaluate the cardiac autophagy status in clinical practice.

### 3.3 Interplay between ROS and cardiac autophagy

Ischemic myocardial tissue not only restores nutrition and oxygen supply, but also accumulates a large amount of reactive oxygen species (ROS). Autophagy is over-activated during reperfusion. Similarly, an *in vitro* hypoxia/reoxygenation (H/R) model showed that autophagy was activated in the initial hypoxic phase and hyperenhanced during the reoxygenation phase [39]. Excessive ROS generation further aggravates cardiac metabolism disturbances, which further causes disordered autophagic flux. In contrast, dysfunctional autophagic flux induced by ROS causes disordered cardiac metabolism.

### 3.4 Role of autophagic flux in cardiac metabolic disorders in myocardial ischemia and reperfusion stages

There is a close coordination between metabolic stages and cardiac autophagic flux, with levels of nutrients and metabolites critically inducing or inhibiting flux intensity in response to external stimuli in the metabolic-oxidative stress-signaling pathway-autophagic flux model. Recent studies have shown that energy disorders, oxidative stress-induced autophagy, and mitophagy play critical roles in the onset and progression of various pathophysiological conditions and diseases, particularly myocardial ischemia, and MI/R [40]. Specifically, the beneficial effects of autophagy in MI/R may be related to adenosine triphosphate (ATP) production and cellular homeostasis. In the early stages of ischemia, the activation of autophagy can maintain energy homeostasis, mainly by restoring ATP production, which degrades fat and protein, releasing free fatty acids and amino acids. Then ATP is generated via the tricarboxylic acid (TCA) cycle to compensate for the energy crisis during myocardial ischemia, thus allowing energy to be restored and cardiomyocytes to survive [40]. Glucose deprivation-induced oxidative stress stimulates aggresome formation and autophagy in cultured cardiomyocytes [41]. Therefore, we have summarized several signaling pathways that are closely linked to oxidative stress, cardiac metabolism, and autophagic flux.

#### (1) PI3K-Akt-mTORC1 axis and other Phosphoinositol 3-kinasen (PI3K) related pathway

Three different classes of (PI3K) are present in vertebrates, class I, II, and III; the integral role of PI3K family-related pathways in the control of cell growth, proliferation, survival and metabolism, particularly the autophagic network, has been previously elucidated [42]. Class III PI3K/Beclin-1 signaling pathway is regulated by ROS. Yan et al. found that in H/R-induced myocardial injury, tissue factor pathway inhibitor (TFPI) inhibited the class III PI3K/Beclin-1 signaling pathway by reducing intracellular ROS levels, thus protecting cardiomyocytes from H/R injury [43]. In conclusion, under normal nutritional conditions, class I PI3K regulates a well-nourished signaling cascade that drives the production of the second messenger PIP-3 and activates serine/ threonine protein kinase (AKT) with the assistance of phosphatidylinositol-dependent protein kinase 1 (PDK1). Notably, the mammalian rapamycin complex 1 (mTORC1) is recognized as a master regulator of autophagy. Inhibition of tuberous sclerosis complex-1 or 2 (TSC1/2) activates mTORC1, which binds to and phosphorylates the mammalian autophagy initiating kinase Unc-51-like kinase 1/2 (ULK1/2) complex [42]. Importantly, mTORC1 plays a central role in regulating the processes that increas the production of proteins, lipids, and nucleotides, and therefore controls the balance between anabolism and catabolism in response to diseases, such as myocardial ischemia and reperfusion [44].

In addition to the PI3K-Akt-mTORC1 axis, class III PI3K usually includes obligate complexes that can phosphorylate phosphatidylinositol (PI) to form phospholipid meriositol 3 phosphates (PI3P), which uses proteins in the cytoplasm to form autophagosome membranes. Class III PI3K can also form a complex with Beclin-1, which plays an important role in initiating autophagosomes formation [45]. In addition, PI3K activates serum and glucocorticoid-induced kinase-1 (SGK1), which in turn activates glycogen synthase kinase 3β (GSK3β), promoting cardiomyocyte survival during hypoxic injury [46]. Class II PI3K is thought to be a generative pool of phosphatidylinositol 3-phosphate (PtdIns3P) that promotes autophagy by coordinating with the class III PI3K-related cascade [47].

#### (2) Beclin 1 related pathway

The Beclin 1 core complex includes Beclin 1, Vps15, Vps34, and possibly Ambra1. Numerous proteins that interact with Beclin 1 induce or inhibit autophagy. Members of the anti-apoptotic family (e.g., Bcl-2, Bcl-XL and Mcl-1) are also important negative regulators of autophagy through the inhibitory interaction of their BH3 binding groove with the BH3 structural domain of Beclin 1 [48]. It has been reported that the Luhong Formula and Hydroxysafflor yellow A can inhibit cardiac autophagy by suppressing HIF1α-mediated ROS production, as evidenced by a decrease in Beclin1 protein levels (i.e., ROS regulates the Beclin1 related pathway) [49]. Furthermore, ROS generated by myocardial ischemia or reperfusion injury activates NF-κB, upregulates Beclin 1 expression, promotes autophagic activity, and ultimately leads to an aggravated cardiac injury in MI/R [50].

#### (3) AMPK pathway

AMP-activated protein kinase (AMPK), a well-known energy sensor in cardiomyocytes [51], is driven by a reduction in the energy charge of the adenylate system (biological functions of intracellular AMP, ADP, and ATP concentrations: (1/2 C_ADP_+C_ATP_)/(C_AMP_+C_ADP_+ C_ATP_). Under conditions of glucose deprivation and AMPK accumulation, AMPK initiates autophagy by directly promoting the separation of ULK1 from mTORC1 via phosphorylation [52]. In myocardial ischemia and reperfusion, excessive ROS accumulation triggers a chronic autophagic response, leading to increased apoptosis and reduced myocardial function, which may occur via AMPK/mTOR [41]. Additionally, AMPK regulates Forkhead box O (FOXO) 3 -mediated hypoxia-induced autophagy in cardiomyocytes [53]. AMPK plays a role in metabolic regulation [54], and its function includes the regulation of fuel supply and energy-generating pathways in response to the metabolic needs of the heart during myocardial ischemia and reperfusion [55].

#### (4) MAPK pathway

Mitogen-activated protein kinase (MAPK), activated by ROS, has been shown to play a key role in coordinating cellular responses to various stimuli and in linking autophagy to inflammation and oxidative stress [56]. Furthermore, many promising agents exert their cardioprotective effects via the MAPK pathway [57-59]. Intriguingly, mTOR has also been reported to be a downstream regulatory signal of MAPK, which is involved in the autophagy-inhibitory effect of the MAPK pathway [60]. Conventional MAPKs are divided into ERK1/2, JNK, and p38, which play key roles in regulating autophagy and myocardial injury. Notably, AMPKs are also considered key regulators of cardiac metabolism during myocardial ischemia and/or reperfusion, which are discussed in detail below.

##### 1) JNK regulatory pathway

The c-Jun N-terminal kinase (JNK), a member of the MAPK subfamily, is essential for cell proliferation and apoptosis. Under normal conditions, JNK is located in the mitochondria and cytoplasm/nucleus; however, when myocardial ischemia and/or reperfusion occurs, JNK is phosphorylated in the cytoplasm and nucleus, and activated JNK is transported to the outer mitochondrial membrane, thus triggering B-cell lymphoma 2 (Bcl2)-regulated autophagy and aggravating myocardial injury [61]. Moreover, some studies have shown that ROS accumulation during myocardial ischemia activates JNK and blocks autophagic flux, leading to caspase-dependent apoptosis and aggravation of myocardial injury [53]. Blockade of the JNK pathway can inhibit the harmful effects of H/R injury on mitochondrial function, energy metabolism and redox balance [62]. Moreover, JNK regulates glucose metabolism in various animal models, including obese mice and type 2 diabetes models [63]. The JNK signaling pathway may be a potential therapeutic target for metabolic disorders, particularly myocardial ischemia and/or reperfusion [64].

##### 2) ERK1/2 regulatory pathway

The extracellular signal-regulated protein kinase1/2 (ERK1/2) pathway, a regulator of glucose transport and metabolism, is an important signaling cascade that regulates cardiomyocyte homeostasis and is an important step in autophagy [65]. Under H/R conditions, the activation of ERK stimulates autophagy, which is dysregulated and in turn leads to cardiomyocyte dysfunction [53]. Furthermore, deficiency of sprouty-related EVH1 domain protein 2 (SPRED2), an intracellular repressor of extracellular signal-regulated kinase (ERK) signaling that is prominently expressed in the human heart, leads to impaired autophagy and heart failure [66].

##### 3) p38 regulatory pathway

The p38 signaling pathway is a MAPK pathway that plays an important role in phosphorylating nuclear transcription factors and regulating gene expression. Increased P38 phosphorylation in MI activates autophagy and exacerbates myocardial injury [67]. In addition, palmitate (PA) enhances ROS production and activates p38 and JNK, thereby stimulating autophagy and causing myocardial dysfunction [68]. Wang et al. found that asiatic acid protects cardiomyocytes from ROS-mediated autophagy during MI/R injury via the p38-MAPK/Bcl-2 signaling pathway [5].

#### (5) p53-mediated pathways

p53, a transcription factor that serves as a master sensor for cellular stress, has been reported to have a crucial impact on pathological progression following MI, including autophagy [69], ferroptosis [70], and apoptosis [71], in addition to its importance in all major cancers. Interestingly, p53 also participates in cardiac metabolism [72]. Autophagy is reflected in the genetic and pharmacological manipulation of p53, which attenuates MI/R damage by inhibiting autophagic clearance [73]. In contrast, other studies have shown that mitochondrial quality control is compromised after MI due to the activation of p53 [74]. For example, p53 upregulates TIGAR (a TP53-induced glycolysis and apoptosis regulator) and reduces Bnip3 activation and cardiomyocyte mitophagy by inhibiting ROS signaling, resulting in impaired mitochondrial integrity and ultimately exacerbating myocardial injury [74]. Intriguingly, the dual effect of p53 on autophagy appears to depend on its subcellular localization. Autophagy is induced in a nuclear p53-dependent/non-dependent manner: on the one hand, p53 activates AMPK and/or antagonizes mTOR, and on the other hand, p53 directly promotes damage-regulated autophagy modulator (DRAM) at the transcriptional level, thereby promoting autophagosome-lysosome fusion [75], whereas cell membrane p53 may inhibit the activation of autophagy, subsequently leading to mitochondrial dysfunction and cardiomyocyte death [76].

#### (6) Endoplasmic reticulum (ER) stress

ER stress is mediated by three pathways: the inositol-requiring enzyme-1(IRE-1), PRKR-like ER kinase (PERK) and activating transcription factor 6 (ATF6) pathways, all of which can activate autophagy. An increase in ROS can promote ER stress leading to autophagy activation, and the activation of ER stress can, in turn, cause ROS production, which creates a vicious feedback loop [77, 78]. Vascular endothelial growth factor A (VEGF-A) activates the ROS-ER stress-autophagy axis in vascular endothelial cells and induces compensatory angiogenesis. Moreover, VEGF-A trigger ER stress-activated autophagy mainly through the upregulation of IRE-1 [79]. Additionally, ER stress induces metabolic alterations in cardiomyocytes, characterized by a shift from fatty acid to glycolytic substrate consumption, and contributes to the impairment of energy metabolism reported in most cardiac diseases, particularly in myocardial ischemia and/or reperfusion [80].

#### (7) Thioredoxin-interacting protein (TXNIP)

As a pro-oxidant protein, TXNIP negatively regulates thioredoxin activity and its antioxidant function, which have been reported to be induced during ischemia and further increased during reperfusion. In MI/R, activation of TXNIP increases autophagosome formation by upregulating Redd1 [81], and inhibits the clearance of autophagosomes by inducing ROS, thus aggravating MI/R injury [81]. A novel link between GLUTs and TXNIP was demonstrated, highlighting the fundamental regulatory mechanisms of glucose homeostasis in the heart [82].

#### (8) Transient receptor potential mucolipin 1 (TRPML1)

Increased ROS production in MI/R activates the lysosomal cation channel TRPML1, which releases lysosomal zinc into the cytoplasm and blocks the cardiac autophagic flux by disrupting the fusion of autophagosomes and lysosomes. Inhibition of autophagy mediated by TRPML1 disrupts mitochondrial turnover and leads to further release of ROS, which directly leads to cardiomyocyte death after MI/R (i.e., ROS linked by TRPML1 and autophagy inhibition form a vicious cycle, leading to myocardial damage) [4]. Additionally, lysosomal TRPML1 regulates cellular metabolism in breast cancer cells, as demonstrated by decreased glycolysis and ATP production [83].

## 4. Cardiac metabolism during myocardial ischemia and reperfusion stages

### 4.1 Overview of cardiac metabolism and energy production

To maintain the heartbeat and contractile function, the heart consumes more energy than any other organ. To achieve this, the heart metabolizes various fuels (fatty acids, glucose, and amino acids) to produce ATP. Apart from producing ATP, cardiac metabolites also constitute key metabolic regulators that have profound effects on cardiac structure and function and are closely related to oxidative stress [84]. Notably, changes in cardiac metabolism, such as changes in some glucose modulators, have been demonstrated to be an effective strategy for improving cardiomyocyte survival in patients with myocardial ischemia or MI/R [85].

### 4.2 ROS-mediated modulation of cardiac metabolism

Myocardial ischemia is associated with changes in metabolic pathways [86]. During MI/R paroxysms, diabetes or stress hyperglycemia induces glucose metabolism disorders and the accumulation of energy substrates, which can promote cardiac oxidative stress through a variety of mechanisms. Under hyperglycemic conditions, NOX2 activation increased ψm and decreased mitoHKII activity led to ROS production. During MI/R, abnormal FA metabolism is indicated by the accumulation of long-chain acylcarnitines in the ischemic mitochondria. During ischemia, triglyceride storage in the myocardium decomposes owing to a sharp reduction in blood flow, but FAs metabolism does not match oxidation in the mitochondria, resulting in the accumulation of long-chain acylcarnitines in the mitochondria. Because stimulated synthesis and CPT2-dependent-oxidation rates are extremely reduced, long-chain acylcarnitines accumulate in ischemic mitochondria. In addition, the transfer of long-chain acylcarnitines from the intermembrane space to the mitochondrial matrix is hampered by the relative lack of mitochondrial carnitine. Under pathological conditions such as hypoxia/ischemia, oxidative phosphorylation is blocked, and the increased generation of α- ketoglutarate drives the accumulation of succinate. During reperfusion, succinate is rapidly oxidized by succinate dehydrogenase to produce ROS. Succinate accumulation during ischemia may play a beneficial role; however, during reperfusion it leads to ROS production and exacerbates MI/R injury [87].

### 4.3 Alterations in cardiac metabolism during myocardial ischemia and reperfusion

**Figure 2.**
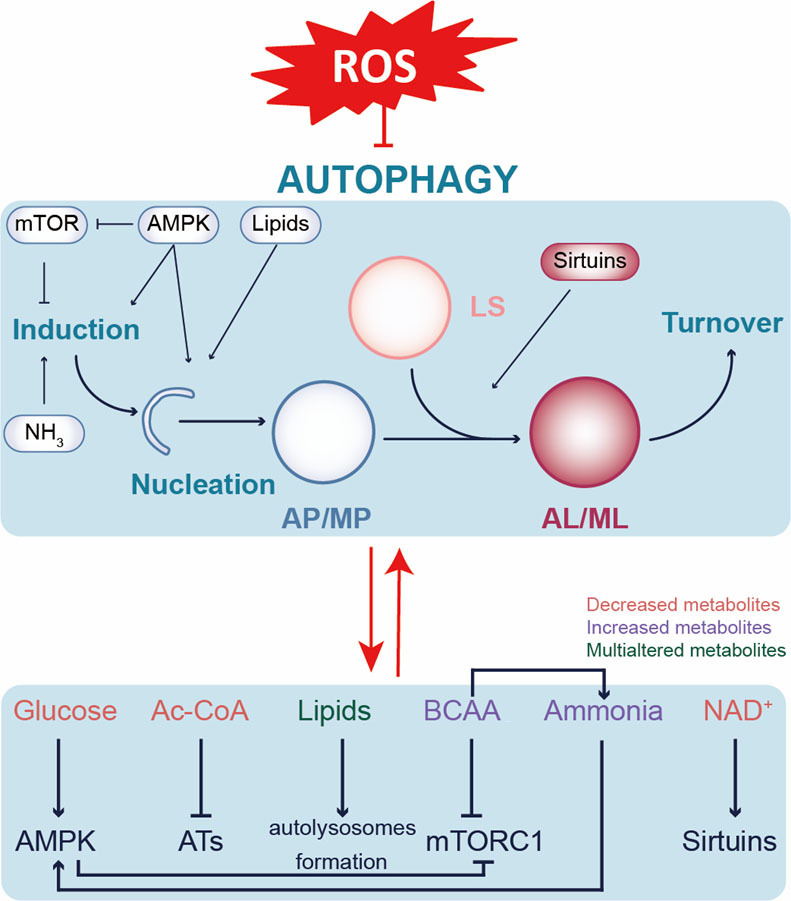
**Metabolic regulatory network of ROS-related autophagic flux**. Ac-CoA: acetyl-CoA; AMPK: 5’ AMP-activated protein kinase; AL/ML: autolysosome/mitolysosome; AP/MP: autophagosome/mitophagosome; ATs: acetyltransferases; BCAA: branched chain amino acids; LS: lysosome; mTORC1: mechanistic target of rapamycin complex 1.

As the metabolomic approach reflects the combined effects of genetics, environment and physiology and represents the ultimate response of the organism to internal and external stimuli, metabolome information is closer to a meaningful pathological/physiological phenotype [88]. Furthermore, there is a close link between cardiac metabolism and autophagic flux, with the levels of nutrients and metabolites critically inducing or inhibiting flux intensity in the metabolite-sensor-regulatory pathway-autophagic flux model (Fig. 2.). During MI/R, autophagy is enhanced during both the ischemia and reperfusion phases. Treating myocardial ischemia or MI/R by modulating autophagy is currently a popular approach; hence, modulating autophagy by modulating cardiac metabolism occurs at the right moment in myocardial ischemia or MI/R injuries.

**Figure 3.**
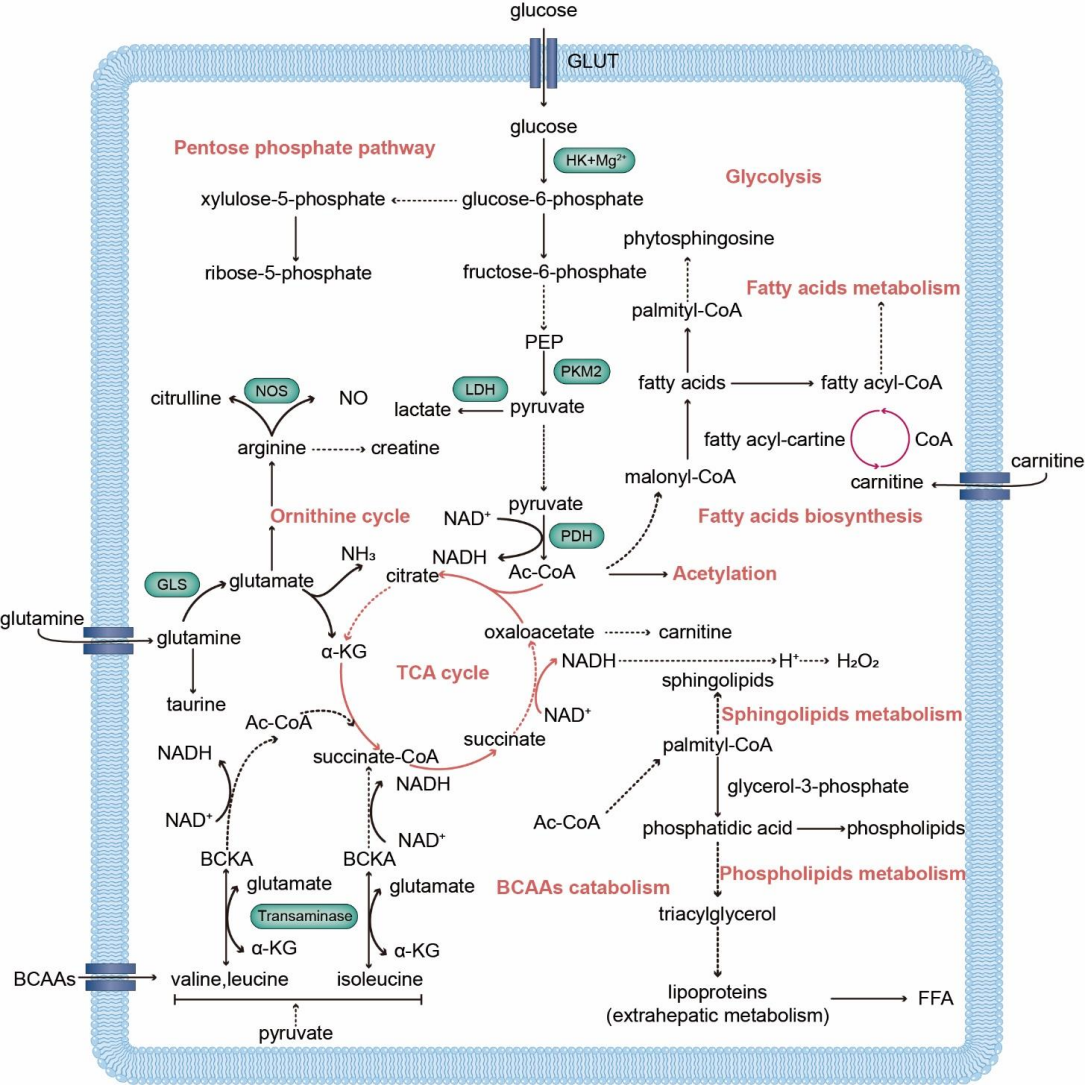
**Summary of altered metabolic pathways in ROS-related autophagic flux during MI and MI/R injury based on metabolomics**. α-KG: α-ketoglutarate; Ac-CoA: acetyl-CoA; BCAA: branched chain amino acid; BCKA: branched-chain keto acids; FFA: free fatty acids; GLS: glutaminase; GLUT: glucose transporter; HK: hexokinase; LDH: lactate dehydrogenase; NO: nitric oxide; NOS: nitric oxide synthase; PEP: phosphoenolpyruvate; PKM2: pyruvate kinase isozyme type M2.

Notably, autophagy has profound effects on cardiac metabolism, and metabolic alterations play a role in autophagy in some MI/R models. From this metabolomic information, metabolomics offers new ideas and methods for exploring the mechanisms of MI/R injury onset and progression more effectively, offering new possibilities for personalized treatment. Studies focusing on the underlying mechanisms of metabolite regulation of autophagy during MI/R are relatively limited; therefore, we summarized the metabolic pathways based on altered metabolomics (Fig. 3 and Table 2). These findings provide new insights for further studies on the metabolite regulated autophagy patterns in MI/R. Establishing metabolomics as a general approach in autophagy research will provide scientists with a biological focus on autophagy an unprecedented opportunity to explore the metabolome of biomarkers of disease states and to understand the diversity of autophagic metabolic pathways in human diseases, with a particular focus on myocardial ischemia and/or reperfusion.

#### (1) Glycolysis

Glucose is a key metabolite in the regulation of autophagy, participating in oxygen metabolism in mitochondria and producing energy in the form of ATP through oxidative phosphorylation [89]. Xie et al. found that dichloroacetate (DCA) inhibits ROS production during reperfusion, thereby inhibiting ROS-activated autophagy, improving glucose homeostasis, and ultimately ameliorating MI/R injury [90]. At high glucose levels, ATP is converted to cAMP [91], which in turn phosphorylates autophagy-related proteins (e.g., ATG1 and ATG13) and mTORC, thereby inhibiting autophagy. In contrast, phosphorylation is inhibited at low blood glucose levels, thereby activating autophagy [92]. Increased glycolysis protects the ischemic myocardium during myocardial ischaemia and early reperfusion, making it a potentially important metabolic target for the treatment of myocardial ischemia [93]. Therefore, glucose, ATP, AMP, and cAMP levels are well suited to indicate the autophagic capacity of cells in MI/R injury. High levels of these metabolites reflect the inhibition of autophagic flux [92] and regulating autophagy by promoting glycolysis is a suitable approach for the treatment of MI/R injury.

**Table 2:** The relationship between metabolites and autophagy.

	Metabolite	Autophagic Flux Process	Autophagy Pathway	Ref.
**Glycolysis**	G-6-P	N/A	Inhibition of autophagy by HK2- mTOR	[95, 96]
	**Fructose-2,6-diphosphate**	N/A	Induction of autophagy by inactivating phosphorylated (P-)mTOR, P-AMPKα, LC3 and Sirtuin1 and activating P62	[97]
	**Lactic acid**	Promotes lysosome acidification and thus vesicles maturation and activation of some proteases during autophagy	SIRT5 induces deacetylation of LDHB, leading to the autophagy overactivation	[94]
**Lipid metabolism**	PE	Phagosome degradation	Activation of the autophagy initiation as an autophagic membrane anchor for autophagy-associated protein LC3	[104, 105]
	**PA**	N/A	Regulation of JNK1-Beclin1-PIP3 pathway	[110]
	**OA**	Elicits the association of the autophagosome-linked MAP1LC3 with the Golgi apparatus	N/A	[110]
	**Arachidonic acid**	N/A	Inhibition of PI3K/Akt and Rho/ ROCK pathways	[106]
	**Ketone bodies**	N/A	Gpr109a - AMPK - NRF2 pathway	[111]
**Amino acid metabolism**	BCAA	N/A	Activation of mTORC1	[122]
	**Glutamine**	Inhibit autophagosome formation	1) Activation of mTORC1 through Arf1 2) Elevation of ammonia level promoted the expression of mitochondrial autophagy markers (BNIP3, PINK1 and Parkin	[125]
	**Arginine**	Inhibit autophagosome formation	1) NO activation of AKT - mTORC1; 2) NO affects the activation of JNK1 through S-nitrosylation Signals can be transmitted to mTORC1 through the known Rag GTPase signaling pathway	[217]
**Other endogenous substances**	NAD^+^	N/A	endogenous abundance overload, artificial precursors repletion, Genetic or Xuanzuo all Induce Autophagic process by activing TFEB pathway.	[132]
	**H_2_S**	N/A	H_2_S restores autophagic flux by activating AMPK pathway in reperfusion heart, ameliorate thyroxine-induced fibrosis by PI3K/AKT pathway, protects aged heart via AMPK-mTOR pathways and increases PINK1-Parkin-mediated mitophagy plausibly by S-sulfhydration of corresponding deubiquitinase.	[129, 130]

Autophagy is regulated by enzymes and molecules involved in glycolysis and the metabolism of intermediates such as palmitic acid (PA), lactic acid, hexokinase (HK), and 6-phosphofructokinase (PFK) [94]. HK is a key enzyme in the first step of the glycolytic pathway that converts glucose into glucose 6-phosphate. HK2 promotes autophagy by inhibiting mTOR during glucose deprivation. In contrast, in the presence of glucose, this binding is inhibited by glucose 6-phosphate (G6P), which is catalyzed by HK2 [95]. Autophagy can also inhibit glycolysis by selectively degrading HK2 in cells with high autophagic flux [96]. The family of phosphofructokinases/fructose diphosphatases (PFKFBs) controls the conversion between fructose-6-phosphate and fructose-2,6-bisphosphate, and according to previous studies, downregulation of PFKFB3 can induce autophagy through inactivation of phosphorylated P-mTOR, P-AMPK α, LC3, and SIRT1, and activation of P62 [97].

#### (2) Lipid metabolism

According to previous reports, lipids and lipid-derived molecules are significantly altered in cardiovascular diseases such as MI/R [98]. The metabolism of linoleic acid and glycerophospholipids is the most important pathway reflecting the late response to MI/R injury. Levels of certain free fatty acids (FFA), such as arachidonic acid, docosapentaenoic acid (DPA), eicostrienoic acid, docosahexaenoic acid (DHA), and certain lysophospholipids, increased during the ischemic period but decreased gradually after reperfusion compared with the ischemic period [99]. However, lipids are closely associated with autophagy. The inhibition of autophagic flux can lead to the accumulation of nuclear receptor co-repressor 1 (NCoR1) and inhibition of peroxisome proliferator-activated receptor alpha (PPARα) activity, leading to impaired lipid oxidation [100]. Furthermore, lipids are necessary for autophagosome formation, suggesting that they can induce autophagy [101].

##### 1) Lipids

Autophagy mediates lipolysis, a process regulated by the transcription factors FOXO1, lysosomal acid lipase (LIPA) and TFEB. In the absence of nutrients, upregulation of the transcription factor FOXO1 or LIPA promotes autophagy-dependent LD degradation and subsequent FA release in adipocytes via AMPK-dependent-oxidation. TFEB-mediated peroxisome proliferator-activated receptor gamma cofactor 1-α (PPARGC1α) and PPARα link autophagy to lipolytic metabolic processes. Furthermore, PPAR-induced TFEB activation or microrNA-33-mediated TFEB inhibition may form a feedback loop that regulates lipolysis and oxidation. In addition to transcription factors, phosphoinosin-3 kinase regulates subunit 4 (PIK3R4, also known as VPS15 in yeast), is key in regulating PPARα activation [102].

There are three types of lipids, phospholipids, fats, and sterols, and their metabolism of phospholipids changes significantly during MI/R. According to previous literature, phospholipid catabolism and sphingolipid metabolism are increased and glycerophospholipid metabolism is impaired in animals with MI. The reversal of metabolic changes plays a cardioprotective role in MI/R injury [103]. Li et al. performed a metabolomic analysis of heart tissue based on ultra-high performance liquid chromatography with quadrupole time-of-flight mass spectrometry (UPLC-Q-TOF-MS) to reveal the effect of copper on autophagy in pig hearts. In this study, excessive copper exposure significantly up-regulated phosphorus ethanolamine (PE) levels and down-regulated phosphatidylserine (PS). PE is essential for the activity of various respiratory complexes and plays a key role in the initiation of autophagy by acting as an anchor for the autophagy-associated protein LC3 [104]. Studies have shown that autophagy mediates PS exposure and phagosome degradation, implying that increased PS levels are associated with autophagy [105]. So, phospholipids could play a cardioprotective role by regulating autophagy.

Arachidonic acid, an essential fatty acid, is an agonist of Rho-related protein kinase (ROCK), which activates autophagy and protects the myocardium via the Rho-Rock pathway [106]. Visceral adipose tissue-derived serine protease inhibitor (Vaspin), an adipokine reported to be present in the visceral and subcutaneous adipose tissue of obese individuals, reduces I/R or H/R-induced myocardial apoptosis and is activated by the AMPK-mTOR pathway to activate autophagic flux and restore lysosomal function [107].

##### 2) FFA

Li et al. found that excessive copper exposure can significantly reduce fatty acid levels. Fatty acids are an important energy source, and their reduced levels induce disturbances in mitochondrial energy metabolism, generating large amounts of ROS and leading to autophagy [108]. In general, saturated fatty acids (C15-C18) induce autophagy through the production of PI3P, whereas unsaturated fatty acids induce atypical autophagy [92].

Sun et al. performed UFLC-MS/MS-based targeted metabolomics analysis of metabolites involved in ISO-induced pathogenesis of AMI, and the data showed significant changes in FFA in the plasma and myocardium of AMI rats. PA, oleic acid (OA), linoleic acid and arachidonic acid are the free fatty acids most associated with AMI [109]. Moreover, according to previous reports, PA can induce the activation of JNK1, stimulates the activity of Beclin 1 / phosphatidylinositol 3-kinase downstream of JNK1, and catalyzes the formation of PI3P from the PIK3C3 subunit. OL elicits JNK1 and Beclin1/PIK3C3-independent association of autophagosome-linked microtubule-associated protein 1A/1B light chain 3 (MAP1LC3, known as LC3) with the Golgi apparatus [110]. Arachidonic acid is an agonist of ROCK and can activate autophagy via the Rho-Rock pathway. It is likely that PA, OA, and arachidonic acid exert protective effects on the myocardium by modulating autophagy.

Acetone, acetoacetic acid and beta-hydroxybutyric acid (beta-OHB) are intermediate products of the oxidative breakdown of fatty acids and are collectively referred to as ketone bodies; beta-OHB is the most concentrated ketone body in the blood. The exogenous transport of ketone bodies, particularly β-OHB, may be an appropriate protective strategy against myocardial ischemia [111]. Autophagy promotes the release of free fatty acids, which are oxidized for ketone body biosynthesis, implying that β-OHB biosynthesis can be impaired by blocking autophagy [112]. Various studies have attempted to regulate ketone body metabolism through physiological or nutritional strategies, such as short-term ketogenic diets (KD, 2_4 weeks), long-term KD (19 weeks), β-OHB infusion. Different intervention outcomes were observed. In a study assessing the effectiveness of the KD on cardiac function in MI mice, Stephen C and Kolwicz Jr. found that an increase in serum ketone bodies may inhibit glucose utilization during myocardial ischemia, thereby exacerbating myocardial injury. However, the cardioprotective effects of β-OHB or ketoester (KE) in reperfused or ischemic myocardium can be exerted through improved energy transfer from mitochondria [111]. The improvement in vascular health following interventions such as intermittent fasting and ketogenic diets is not necessarily a secondary effect of improved metabolic indices, but may be due to a direct vasodilatory effect, which is induced by the autophagy of β-OHB via the Gpr109a receptor [113]. Further research is required to evaluate these treatment strategies.

#### (3) Amino acids metabolism

The metabolism of amino acids (AAs) also undergoes a deleterious change that contributes to the vulnerability of the heart to MI/R injury because various AA are carbon sources for cellular bioenergy, maintain cellular redox homeostasis, and participate in signalling pathways. Studies have shown that metabolites associated with AAs clearly regulate autophagy through two main pathways, either non-selectively or by targeting the mitochondria. For example, the inappropriate supply of AAs in the lumen of the lysosome is transferred to the lysosomal surface and activates mTORC1, which has a negative regulatory effect on autophagic flux through the Rag-guanosine triphosphatase (Rag- GTPase) pathway or the ADP-ribosylation factor1 (Arf1) pathway [114]. Specifically, altered signals from AAs are differentially sensed by certain sensors and transduced to downstream transfer stations (Rag GTPases) or Arf1, which are converted to their active forms and are required for mTORC1 activation. In contrast, the lack of intracellular AAs is accompanied by the accumulation of uncharged tRNA species, which promote autophosphorylation of general control nonderepressible 2(GCN2) by AAs in a non-specific manner [115] and subsequently trigger autophagy at the transcriptional level via the eIF2α-ATF4 pathway.

##### 1) BCAA

BCAA cause mitochondrial dysfunction and increase ROS production in the heart, thereby activating AMPK-ULK1 pathway-dependent autophagy, leading to myocardial injury in mice [116]. BCAA catabolic enzymes have been shown to be expressed predominantly in the heart. Although the contribution of this oxidative pattern to energy production is limited, systematic metabolomic studies highlighted the close link between aberrant metabolism of BCAA and defects in cardiac metabolism. Impaired BCAA catabolism significantly interferes with glucose uptake and pyruvate utilization by decreasing the activity of the pyruvate dehydrogenase complex (PHD). This remodelling of the metabolome renders the heart vulnerable to MI/R injury, an unfavourable outcome that can be rescued by restoring BCAA catabolism or by overexpressing the type 1 glucose transporter (GLUT1) in the PP2Cm KO model (the absence of PP2Cm leads to a deficit of BCAA catabolism) [117]. Furthermore, this catabolic deficit directly contributes to the cardiac dysfunction, remodelling and adverse cardiovascular events induced by clinical interventions [118]. In a cohort of deceased patients with AMI, differential network analysis revealed higher levels of isoleucine in the low-risk-score network and higher levels of valine in the high-risk score network [119].

As the concentration of BCAA increases, the corresponding sensor-mTORC1 network is activated and autophagic flux is blocked [120]. Intriguingly, there is evidence that in many cell lines and mice, the cytoplasmic levels of leucine are sufficient to alter the activity of EP300 acetyltransferase (via its Krebs cycle metabolite Ac-CoA, rather than directly), which acetylates raptors, ultimately leading to mTORC1 activation and autophagic/mitophagic flux [121]. In contrast, exogenous Ac-CoA supplementation or EP300 stimulation had little effect on the elevation of LC3-II levels under leucine depletion, further suggesting a role for leucine in regulating autophagy via mTORC1.

Moreover, BCAA have a deleterious impact on other metabolic pathways, which enhances glycolysis and fatty acid oxidation but impede glucose oxidation. Valine, leucine, and their corresponding branched-chain α-keto acid derivatives mechanically exacerbate I/R damage via GCN2-ATF6-PPARα pathway-dependent fatty acid oxidation [122].

##### 2) Glutamine and derivatives

Previously, decreased histidine, glutamine, and glutamate levels were interpreted as a response to MI/R injury and, surprisingly, no significant differences were observed between the exercising and resting hearts [123]. A similar trend was observed in our previous investigations; and fortunately, the prior use of orexin antagonized malignant transformation [124].

As a nitrogen source for the biosynthesis of nucleotides and AA and as a carbon source for bioenergy in the Krebs cycle, glutamine is primarily involved in glutaminolytic metabolism, where it is converted to glutamate and subsequently to alpha-ketoglutamate (αKG) and ammonia. Previous studies have reported that increased levels of ammonia promote the expression of mitotic markers (BNIP3, PINK1 and Parkin) in mammalian cells [125] and have suggested that two pathways are involved in this by-product-induced autophagy: one in which ammonia upregulates a phosphate site on AMPK, inducing autophagy, and the other which occurs in response to unfolded proteins [126]. In addition, glutamate promotes Parkin translocation to the mitochondrial membrane [127].

#### (4) Other endogenous substances

##### 1) H_2_S

Hydrogen sulfide (H_2_S) acts as a biological donor for sulfhydration, is endogenously generated from sulfur-containing AAs catabolism, and plays a crucial role in pathological cardiometabolic alterations.

The interaction between H_2_S and autophagy is complex. H_2_S donors can restore autophagic flux by activating the AMPK pathway in reperfused hearts [128], ameliorating thyroxine-induced fibrosis via the PI3K-AKT pathway [129], and protecting senescent hearts through the AMPK-mTOR pathway. Similarly, exogenous H_2_S administration facilitates Parkin translocation to the mitochondrial membrane and increases PINK1-Parkin-mediated mitophagy plausibly by S-sulfhydration of the corresponding deubiquitinase [130].

##### 2) NAD^+^ and NADH

NAD^+^ and NADH not only support redox reactions and coordinate the activation and elimination of ROS, but also act as cofactors for sirtuins in a unique metabolic circuit, as described above [131].

During MI/R, cardiac NAD^+^ levels are curtailed by endogenous overload, artificial precursor repletion, and genetic or pharmacological manipulation [132]. Furthermore, the intracellular concentration of NAD^+^ affects other metabolites that can strongly induce autophagic processes and reduce cardiovascular vulnerability to ischemia and subsequent reperfusion injury, which may be mediated by TFEB [132]. During the TCA cycle, a reduction in NAD^+^/NADH ratio negatively regulates autophagy, which may be associated with impaired acetyl-CoA levels [133].

## 5. Mitophagy and mitophagic flux in myocardial ischemia and reperfusion stages

Mitophagy is a protective form of autophagy during MI/R injury that can remove dysfunctional mitochondria and prevent inflammatory responses and excessive ROS production, thus protecting cardiomyocytes [21]. Additionally, mitophagy removes damaged, abnormal, or dysfunctional mitochondria and replenishes new mitochondria to meet cellular needs through mitochondrial biogenesis. Therefore, mitophagy contributes to protein homeostasis during MI/R injury [134]. Mitophagy is considered an adaptive cardioprotective response during metabolic crises, and drugs that enhance mitophagy can attenuate cardiomyocyte damage [135].

### 5.1 Mitophagic flux in myocardial ischemia and reperfusion stages

Cardiac contraction is a major aerobic process tccchat relies heavily on a continuous ATP supply to maintain physiological viability and function. Simultaneously, the functional mitochondrial network, which is the main hub for ATP production through cellular metabolism, including, but not limited to, electron transport,oxidative phosphorylation, the TCA cycle, amino acid synthesis and fatty acid β-oxidation, is more susceptible to the hypoxic microenvironment. Reperfusion challenges in cardiac tissues are usually accompanied by impaired cellular energy, excessive production, or release of ROS, swelling, and abnormal permeability, ultimately leading to cellular/mitochondrial death [136]. Protecting mitochondrial structure and function through a several approaches that target mitochondria have been shown to ameliorate reperfusion injury and improve cardiac function [137]. Therefore, the quality control of mitochondria, including size, integrity, morphology, and biological activity, is essential.

**Figure 4.**
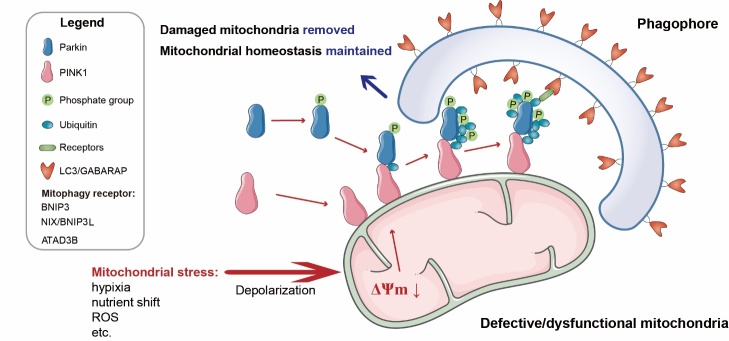
**Overview of mitophagy**. Summary of the three regulatory pathways of mitophagy: PINK1-mediated, BNIP3- and BNIP3L-mediated, and ATAD3B-mediated pathways.

Mitophagy (a contraction of mitochondria and autophagy), capable of selectively clearing redundant and defective mitochondria that accumulate during MI/R, is considered as a cardioprotective mechanism [138]. In this context, mitophagy buffers the cardiotoxic effects of dysfunctional mitochondria. Similarly, the superficial morphological characterization of mitophagy is described in the sequence phagophore-mitophagosome-mitolysosome (PG-MP-ML). A recent study showed that mitophagy involves procedures similar to autophagic flux, including depolarization of damaged mitochondria and decreased membrane potential, formation of mitophagosomes (mitochondria encapsulated by autophagosomes), fusion of mitophagosomes and lysosomes, and the degradation of mitophagosomes by lysosomes [139]. Therefore, mitophagy is a dynamic process similar to autophagic flux, and the concept of mitophagic flux has been proposed in previous studies [140]. Several studies have assessed the potential colocalization between mitochondria and autophagosomes after mitochondrial depolarization by co-transfecting cells with GFP-LC3 and Mito-RFP and showed that mitochondrial phagocytosis could be better assessed with this approach by labeling mitochondria and lysosomes with Mito Tracker Green FM and Lyso Tracker Red, respectively [141]. The most recent assays for mitochondrial phagocytosis are presented in Table 1. Currently, there are many efforts to elucidate the regulatory mechanisms of mitochondria-targeted autophagy, with three dominant mechanisms (Fig. 4).

### 5.2 Crosstalk among oxidative stress, cardiac metabolism and mitophagic flux in myocardial ischemia and reperfusion stages

During myocardial ischemia or MI/R, ROS levels are significantly increased, and endogenous metabolites are significantly altered. Mitochondria are the main sites of ROS production, and damaged mitochondria produce ten times more ROS than normal mitochondria, which further exacerbates mitochondrial dysfunction and creates a vicious cycle [142]. Mitophagy is a defensive metabolic process by which cells adapt to hypoxia. It can effectively remove damaged mitochondria and excess ROS to increase the stability of intracellular mitochondria and reduce MI/R injury [143]. Simultaneously, ROS may regulate some mitophagy-related pathways, disrupt mitochondrial quality, and aggravate myocardial injury [74].

#### 5.2.1 The role of mitophagic flux in cardiac metabolism in myocardial ischemia and reperfusion stages

##### (1) PINK1-mediated pathways

Previous studies have highlighted the pronounced roles of PTEN-induced putative kinase 1 (PINK1) and Parkin via loss-off-function mutants in mitochondrial functionalities: the removal of either PINK1 or Parkin results in a similar phenotype, whereas overexpression of both rescue the defective morphology in mitochondria [144]. In normally functioning mitochondria, PINK1 is transported across the membrane and broken down by an enzyme (mitochondrial rhomboid protease, PARL). During myocardial ischaemia, mitochondria become fragmented and depolarized due to a change in mitochondrial dynamics from fusion to fission [145]. The undesirable mitochondria usually feature a drop in mitochondrial membrane potential (ΔΨm), which is sensed and transduced to Parkin via the autophosphorylation of PINK1. Escaping from the presenilin-associated rhomboid-like protein (PARL), Parkin ubiquitylate proteins are activated in the outer mitochondrial membrane, which is a selective recruitment signal to autophagy receptors on the phagophore membrane. Subsequently, mitophagosomes are translocated to the lysosomes for further elimination and replenishment [146]. PINK1-Parkin mediated mitophagy effectively removed damaged mitochondria and excess ROS to increase mitochondria stability in H9c2 [143].

Phosphorylated mitochondrial Tu translation elongation factor (TUFm) inhibits autophagy, whereas non-phosphorylated TUFm promote mitophagy. PINK1 can transform the pro-mitophagy function of TUFm into an inhibitory function by phosphorylating its conserved serine site. Phosphorylation of PINK1-dependent Ser222 determines the dual role of TUFm in mitophagy, which is an evolutionarily conserved Parkin-independent autophagy pathway [147].

##### (2) BNIP3 & BNIP3L -mediated pathways

Similar to FUNDC1, Bcl-2 adenovirus E1B 19kDa-interacting protein 3 (BNIP3) and Bcl2/adenovirus E1B-interacting protein 3-like (BNIP3L) possess a pivotal motif for selective recruitment to phagophores (BNIP3 interacts with LC3, whereas BNIP3L binds to GABARAPL1) [148], and further facilitate mitochondrial clearance. Both receptors are involved in hypoxia-induced mitophagy and are transcriptionally regulated by hypoxia inducible factor 1α (HIF-1α). At the early stage of MI/R, HIF-1α is increased, which in turn activates downstream protein BNIP3. Importantly, BNIP3 decreases the susceptibility to MI/R injury by directly inducing mitophagic responses at the early stage of reperfusion [149]. However, excessive autophagy mediated by BNIP3 promotes oxygen and glucose deprivation/reoxygenatio (OGD/R)-induced neuronal injury [150]. Zheng et al. found that deferoxamine (DFO) combined with sevoflurane postconditioning (SPostC) can activate HIF-1a, further promote HIF-1/ BNIP3 mediated mitophagy, timely resolve mitochondrial dysfunction, reduce ROS production, avoid the attack of mitochondria-derived ROS on normal mitochondria, ensure the stability of mitochondrial function, and ultimately reduce MI/R injury [142].

##### (3) ATAD3B-mediated pathways

Under oxidative stress, ROS levels in the mitochondria increase, resulting in mtDNA (mitochondrial DNA) damage. The ATAD3B-ATAD3A-MTDNA complex breaks down, and the ATPase family AAA domain-containing protein 3 (ATAD3B) is activated (C-terminal shifts from the mitochondrial inner membrane to the outer membrane), acting as a mitophagy receptor, recruiting LC3, initiating mitophagy, and clearing damaged mtDNA [151]. In addition, ATAD3B is associated with ventricular tachycardia, apex torsion, and cardiac arrest due to antipsychotic-induced QTc interstitial prolongation [152].

#### 5.2.2 The role of cardiac metabolism in mitophagic flux in myocardial ischemia and reperfusion stages

In MI/R injury, many metabolites are associated with mitophagy; for example, tryptophan metabolism is the main method of NAD^+^ synthesis in mitochondrial energy metabolism, and disruption of tryptophan metabolism may affect mitochondrial function abnormalities and the production of ATP, which may lead to an increase in autophagy-related protein expression [108]. Taurine can inhibit PP2Ac methylation, which dependents on S-adenosine methionine (SAM), blocking PINK1-mediated mitophagic flux, thereby maintaining high mitochondrial density, achieving metabolic adaptation, and ultimately blocking the energy required for the polarization of M1 macrophages to glycolysis [153].

Hu et al. analyzed urine based on LC-MS/MS metabolomics and found significant changes in metabolites (adenosine, taurine, β -guanpropionic acid, L-isoleucine, and inositol) before and after Rb1 treatment, suggesting that these metabolites were related to mitophagy. These metabolites were significantly increased after Rb1 treatment, which corrected these metabolic disorders. Simultaneously, Rb1 can regulate mitochondrial disorders by stimulating AMPK and activating the Pink1/Parkin and FUNDC1 pathways. Finally, Rb1 protected against AMI by stimulating AMPKα-mediated mitophagy [154]. Metabolic pathway enrichment analysis revealed that the metabolisms of taurine and sub-taurine, glycine and serine, cysteine, methionine, and purine were the most influential metabolic pathways of Rb1 in the treatment of mice with myocardial ischemic injury.

Furthermore, as an endogenous neurohormone, melatonin is inversely association with the occurrence of heart attacks and plays a key role in regulating MI/R injury, as it can exert cardiac protection not only by direct antioxidant effects or activating the cell membrane or nuclear receptors, which widely expressed in the heart, but also by adjusting the complex associated signaling pathways to regulate various physiological processes; for example, it can protect the heart against MI/R by inhibiting autophagosome formation via the AMPK/mTOR pathway [155] . Melatonin has also been reported to exert cardioprotective effects in MI/R via KH2/PINK1-Parkin pathway-mediated mitophagy [155].

## 6 Therapeutic implications

### 6.1 Potential targets for ROS modulation for cardioprotection via autophagy modulation in myocardial ischemia or MI/R

Some autophagy regulators regulate effects of the redox metabolism of cardiomyocytes and significantly affect the ROS content. Specifically, spermidine improves MI-induced cardiac dysfunction by promoting AMPK/mTOR-mediated autophagic flux and has been shown to significantly inhibit ROS generation in rats with MI [156]. Trimetazidine (TMZ) alleviates MI/R injury by regulating autophagy through the AKT/mTOR and AMPK/mTOR pathways in MI/R H9c2 cells and SD rats. An in vitro study also demonstrated that TMZ administration mitigated oxidative stress and increased antioxidant enzymes by enhancing the CSE/H_2_S pathway activity before H/R in H9c2 cells [157]. The beneficial effects of Hongjingtian injection (HJT) in treating MI/R are partially due to improved mitochondrial function and regulated autophagy, which inhibit cell apoptosis through the AMPK/mTOR pathway. HJT inhibited ROS production and improved mitochondrial function in H_2_O_2_-induced cells by significantly increasing mitochondrial membrane potential and oxygen consumption [158]. APN inhibits cardiomyocyte autophagy induced by excessive ROS by inhibiting the AMPK/mTOR/ ERK-dependent mechanism induced by H_2_O_2_ [159]. APN modulated ROS metabolite levels and increased antioxidant levels in H_2_O_2_ induced adult rat ventricular myocyte injury [159]. The Danshensu/tetramethylpyrazine derivative (DT-010) has cardioprotective effects, which are attributed to the inhibition of autophagy via the AMPK/mTOR/Ulk1 signaling pathway. DT-010 scavenged ROS and alleviated oxidative stress in the t-BHP-induced oxidative injured cell model using the H9c2 cardiomyocyte-like cell line [160]. Irisin activated Opa1-induced mitophagy and protected against cardiomyocyte injury after MI. Treatment with irisin reduced ROS levels in hypoxia-injured cardiomyocytes [161]. Asiatic acid pretreatment protected cardiomyocytes from ROS-mediated autophagy via the p38 mitogen-activated protein kinase/Bcl-2/beclin-1 signaling pathway in MI/R mice [5]. Administration of asiatic acid decreased ROS and malondialdehyde levels, and increased superoxide dismutase activity in OGD-treated cells [5]. In addition, EGCG and hesperidin inhibit excessive autophagy by activating the PI3K/Akt pathway in MI/R rats [42] and play essential roles in myocardial protection through multiple mechanisms which reduce ROS [162, 163]. While melatonin and Danshensu ameliorate myocardial injury by attenuating autophagy via the mTOR pathway, the inhibition of autophagy by melatonin and Danshensu causes them to scavenge ROS [164-166]. In addition, simvastatin, cloxyquin, polydatin, maslinic acid, gastrodin (GAS), and alpha-lipoic acid (LA) reduce myocardial damage by modulating cardiac autophagic flux, all of which decrease in ROS production [167-176].

### 6.2 Strategies to enhance or suppress cardiac autophagy for cardioprotection via metabolism modulation in myocardial ischemia or MI/R

Autophagy causes changes in metabolites of the body, which in turn protects cardiomyocytes by regulating cardiac metabolism, including redox, glucose, lipid, amino acid, and energy metabolism (Table 3).

**Table 3:** Effects of cardiac autophagy regulators on metabolism in myocardial ischemia and reperfusion stages

Agent	Regulation of autophagy			Effect on myocardial metabolism		
	Model	Changes in autophagy	Ref.	Model	Metabolic change	Ref.
Spermidine	MI rat	AMPK/mTOR-mediated autophagic flux↑	[156]	MI rat	ROS↓	[156]
Sdiponectin	H_2_O_2_ induced adult rat ventricular myocytes injury	AMPK/mTOR/ERK-dependent autophagosome formation↓	[159]	H_2_O_2_ induced adult rat ventricular myocytes injury	Antioxidant levels↑, ROS↓	[159]
Simvastatin	HF rat;cardiomyocyte injury H9c2 cells; MI/R mice	Mitophagy↑ Akt/mTOR-macroautophagy↑	[167]	MI/R mice	ROS↓	[169]
Cloxyquin	MI/R adult cardiomyocytes	autophagy↑	[176]	MI/R adult cardiomyocytes	ROS↓	[176]
Irisin	MI Primary cardiomyocytes	Opa1-induced mitophagy↑	[161]	MI Primary cardiomyocytes	ROS↓	[161]
Danshensu/tetramethypyrazine derivative	MI/R H9c2 cells	AMPK-mTOR-Ulk1-mediated autophagy↓	[160]	t-BHP-induced oxidative H9c2 injured model	ROS↓	[160]
Danshensu	MI/R rat	Autophagy↓	[166]	MI/R rat	ROS↓	[145]
Trimetazidine	MI/R H9c2 cells and SD rats	AKT/mTOR and AMPK/mTOR mediated autophagy↑	[157]	HR H9c2 cells	Oxidative stress ↓antioxidant enzymes↑	[157]
Polydatin	H/R rat	Autophagy ↑	[170]	MI/R rat	ROS↓	[170]
Hesperidin	MI/R rat	PI3K/Akt/mTOR-mediated autophagy↑	[177]	MI rat	Antioxidant status↑	[178]
Alpha-lipoic acid	MI/R rat	Autophagic flux↓	[171]	MI/R rat	Mitochondrial ROS production↓	[171]
Naringin	MI/R rat	Autophagy↓	[172]	MI/R rat	Anti-oxidative capacity↑	[172]
Melatonin	MI	Autophagy↓	[164]	MI	Intracellular ROS generation↓	[164]
Maslinic Acid	MI/R rat	Autophagic flux↑	[173]	MI/R rat	ROS↓	[174]
Gastrodin	MI/R mice	Autophagic flux↑	[175]	MI/R mice	ROS↓	[175]
Hongjingtian injection	MI/R mice	AMPK/mTOR-mediated autophagy↓	[41]	MI/R mice	ROS production↓	[41]
Rmifentanil	H/R H9c2 cells	Autophagic flux↑	[179]	MI rat	Lipid peroxidation↓	[179]
Asiatic acid	MI/R mice	p38 mitogen-activated protein kinase/Bcl-2/beclin-1 -mediated autophagy↓	[5]	MI/R mice; deprivation/reperfusion (OGD)-treated cells	Glycogen breakdown↓glucose, and lactate concentrations↑	[5]
Spermidine	MI rat	AMPK/mTOR-mediated autophagic flux↑	[156]	HF	Lipid accumulation↓	[180]
Trimetazidine	MI/R H9c2 cells and SD rats	AKT/mTOR and AMPK/mTOR-mediated autophagy↓	[157]	H/R H9c2 cells; MI/R rat	Shifting the cardiac substrate preference from fatty acid oxidation to glucose oxidation resulting from 3-ketoacyl coenzyme A thiolase (3-KAT) inhibition	[157]
Metformin	MI/R mice	Cytoplasmic AMPKα1- and nuclear AMPKα2-mediated autophagy autophagic flux↑	[181]	MI/R mice	Fatty acid oxidation↑ and glucose utilization↓	[182]
Aldehyde Dehydrogenase 2	MI/R mice	AMPK/mTOR-mediated autophagic flux during ischemia↑, AMPK/mTOR and AKT/mTOR-mediated autophagy↓ during the reperfusion phase	[183]	MI/R rat	Toxic aldehydes (such as 4-HNE) and its adducts↓	[183]
Alliin	MI/R mice	Autophagy↑	[135]	MI/R mice	Plasma thiobarbituric acid reactive substances and hydroperoxide↓;triglycerides, low-density lipoprotein and non-esterized free fatty acid↓	[135]
Hesperidin	MI/R rat	PI3K/Akt/mTOR pathway-mediated autophagy↓	[42]	MI rat; doxorubicin cardiotoxicity	Lipid peroxidation↓	[178]
Bauhinia championii flavone	MI/R rat	PI3K/Akt-dependent autophagy↓	[185]	MI/R rat	Myocardial energy metabolism↑	[185]
3-methyladenine	Mouse embryonic fibroblasts (MEFs), L929 cells, HEK293T cells, and HeLa cells	Class I PI3K and class III PI3K -mediated autophagy↓	[186]	MI/R rat	ATP synthesis capacity↓	[187]
Cilostazol	MI/R rat	TFEB-mediated autophagy↑	[188]	MI/R rat	ATP↓	[189]
Hydroxysafflor Yellow A	MI/R rat	AMPK-mediated autophagy↑	[190]	H/R rat	ATP↑	[49]
Irisin	MI Primary cardiomyocytes	Opa1-induced mitophagy↑	[161]	MI Primary cardiomyocytes	ATP↑	[161]
Dnshensu	MI/R rat	mTOR-mediated autophagy↓	[166]	MI/R rat	ATP↑	[165]
Hongjingtian injection	MI/R mice	AMPK/Mtor-mediated autophagy↓	[41]	MI/R mice	ATP↑	[41]

#### 6.2.1 Effects of cardiac autophagy regulators on glucose and lipid metabolism

Some autophagy regulators can effectively protect cardiomyocytes by regulating myocardial injury-induced disordered glucose and lipid metabolisms *in vivo*. Hesperidin, remifentanil (RPC) and spermidine regulate cardiac autophagic flux, leading to changes in lipid metabolism. Specifically, hesperidin inhibits excessive autophagy in MI/R rat [158, 177]. A 7-day hesperidin pretreatment inhibited lipid peroxidation and increased antioxidant status in an iso-induced model of MI, in combination with doxorubicin-induced cardiac dysfunction [163, 178]. RPC can protect cardiomyocytes against H/R injury by enhancing autophagic flux and attenuating cardiac dysfunction, lipid peroxidation, and immune disorders in rats with isoproterenol-induced myocardial injury via inhibition of the JNK/NF-KB p65 pathway [179]. Spermidine improved MI-induced cardiac dysfunction by promoting autophagic flux, which significantly inhibited the generation of ROS in rats with MI [156]. Another study showed that spermidine reduced lipid accumulation and atherosclerotic plaque formation in heart failure (HF) [156, 180]. Asiatic acid pretreatment protected cardiomyocytes from ROS-mediated autophagy not only by decreasing the levels of ROS,but also by effectively suppressing MI/R-induced glycogen breakdown and inhibiting the elevation of plasma glucose and lactate concentrations [5].

TMZ alleviates MI/R injury by regulating autophagy. At the cellular level, TMZ exerts anti-ischemic effects on the heart by shifting the cardiac substrate preference from fatty acid oxidation to glucose [157]. Metformin had the dual effects of promoting both cytoplasmic AMPKα1- and nuclear AMPKα2-related signaling to improve autophagic flux and restore cardiac function in MI/R mice [181]. When metformin was administered to MI/R mcie, the activation of AMPK in the early stages of reperfusion promoted fatty acid oxidation and thus inhibited glucose utilization, leading to myocardial damage [182]. The cardioprotective effect of metformin appears to be closely related to its hypoglycemic effects.

Aldehyde dehydrogenase 2 (ALDH)2 plays a dual role in I/R. During ischemia, it activates AMPK and inhibits mTOR, thereby increasing cytoprotective autophagy, while during reperfusion, ALDH 2 inhibits AMPK and activates Akt, thereby activating mTOR,inhibiting AkT-dependent autophagy [183]. ALDH2 reduces MI/R damage by scavenging toxic aldehydes (such as 4-HNE) and their adducts [183]. Alliin (ACSO) alleviated MI/R injury by promoting autophagy. ACSO treatment reduced the levels of triglycerides, low-density lipoproteins and non-esterized free fatty acids [135]. Simultaneously, the increase in the plasma levels of thiobarbituric acid reactive substances and hydroperoxide in mice with myocardial-ischemia was also suppressed by ACSO administration [135, 184].

#### 6.2.2 Effects of cardiac autophagy regulators on energy metabolism

Some autophagy regulators effectively protect cardiomyocytes by regulating energy metabolism to increase ATP production. Bauhinia championii flavone (BCF) exerts protective effects against MI/R by inhibiting apoptosis and excessive autophagy in a PI3K/Akt-dependent manner. BCF also scavenges free radicals and improves myocardial energy metabolism following H/R injury [185]. Three-methyladenine (3-MA), an autophagy inhibitor, has been used in preclinical studies of a variety of diseases to verify the regulatory effect of drugs on autophagic flux. For example, Jianjie Zheng used 3-MA as an autophagy inhibitor as a control to study the mechanism of miRNA-30e reducing MI/R injury through autophagy [186]. Furthermore, 3-MA in the MI/R rats decreased the mitochondrial ATP synthesis ability in the MI/R rats [187]. Cilostazol protects against MI/R injury by regulating autophagy, lysosome, and apoptosis in a rat model of MI/R injury, and partially by increasing the transcriptional activity of TFEB [174, 188]. The use of cilostazol significantly decreases ATP and increases SOD levels in Wistar rat cardiomyocytes after MI/R [189], and hydroxysafflor yellow A (HSYA) activates AMPK to improve autophagy [190]. A study reveals that HSYA could enhance the viability, prevent apoptosis, restore the ∆Ψm loss, and promote the ATP content in H/R treated cardiomyocytes [190]. In addition, irisin, EGCG not only inhibit redox metabolism and reduce ROS generation but also activate myocardial energy metabolism and increase ATP generation in vivo by regulating cardiac autophagy [161, 162, 165, 166].

### 6.3 Metabolic interventions for cardioprotection via autophagy modulation against myocardial ischemia or MI/R

Investigations have confirmed that drugs involved in myocardial metabolism have a regulatory effect on autophagy during the myocardial ischemia and reperfusion stages (Table 4).

#### 6.3.1 Effects of redox regulator on autophagy

Some drugs reduce ROS generation by regulating REDOX metabolism, thereby regulating the myocardial autophagic flux and reducing myocardial damage. Alpha2 adrenoceptor agonist dexmedetomidine (Dex) not only significantly reduced the generation of ROS, but also decreased malondialdehyde content and increased the antioxidant signal [191]. Dex upregulated the phosphorylation of Beclin1 at S295 by activating the PI3K/Akt pathway and reducing the interactions of the Atg14L-Beclin1-Vps34 complex, thereby inhibiting autophagy and protecting against MI/R injury [192]. In H9C2 cells, pretreatment with puerarin, one of the major active components of puerarin, reduced ROS levels and increased GSH and ATP levels to control the abnormal metabolism produced by IR cardiomyocytes [193]. Ferulic acid (FA) can increase the activity of the antioxidant enzymes superoxide dismutase, catalase, and glutathione peroxidase; decrease malondialdehyde levels; ameliorate the production of reactive oxygen species; and promote the generation of adenosine triphosphate in MI/R rats [194]. FA pretreament in MI rat H9c2 could protect H9c2 from apoptosis by enhancing autophagy [195]. Other ROS inhibitors such as metformin, resveratrol, vitamin D, salvianolic acid B, asiatic acid, empagliflozin, hongjingtian injection (HJT), vitexin, puerarin and melatonin reduce ROS production [196-202] and regulate myocardial autophagic flux in myocardial injury [158, 190, 203-209]. Additionally, sufentanil increases the activitiy of glutathione peroxidase and superoxide dismutase in a myocardial injury model [210, 211], which can reverse the oxidative metabolic shift, both of which can lead to changes in autophagy and a reduction in the degree of myocardial damage [212, 213].

**Table 4:** Effects of cardiac metabolic regulators on autophagy.

Agent	Effect on myocardial metabolism			Regulation of autophagy		
	Model	Metabolic change	Ref.	Model	Changes in autophagy	Ref.
Metformin	MI/R mice	ROS↓	[196]	MI rat in vivo and OGD-induced H9c2 in vitro	AMPK mediated autophagic flux↑	[181]
Resveratrol	MI/R rat	ROS↓	[199]	MI/R rat	MEKK1/JNK mediated autophagy↓	[202]
Vitamin D	H/R H9c2 cells	ROS↓	[207]	H/R-induced H9c2 injury	BNIP3/LC3B↓, autophagy↓	[207]
Salvianolic acid B	MI/R rat and cardiomyocyte cell line AC16	ROS↓	[200]	MI/R Murine myocardial cells that had undergone primary culture were induced by MI/R	miR-30a-mediated autophagy↓	[209]
Asiatic Acid	MI/R in vivo and a cell model of OGD/R in vitro	ROS↓	[5]	MI/R mice	ROS-mediated autophagy ↓	[5]
Empagliflozin	MI/R mice	ROS↓	[201]	MI/R mice	ER stress-induced autophagy↓	[208]
Hongjingtian injection	H_2_O_2_-induced H9c2 injury	ROS↓	[41]	MI/R C57BL/6 mice	ROS-mediated autophagic flux↓	[41]
Vitexin	H/R -induced H9c2 injury	ROS↓	[198]	MI/R rat	Autophagy↓	[246]
Puerarin	MI/R H9c2 cells	ROS↓	[243]	H/R-induced H9c2 injury	ANRIL↑, autophagy↓	[243]
Melatonin	H/R H9c2 cells	ROS↓	[247]	MI/R rat	Excessive mitophagy and autophagy↓	[155]
Sufentanil	MI/R rat	The activities of glutathione peroxidase (GSH-Px) and superoxide dismutase (SOD)↑	[210]	MI/R rat	Autophagy↓	[212]
Dexmedetomidine	MI/R-induced H9c2 injury	Superoxide dismutase and plasma glutathione peroxidase activities↑, ROS↓	[191]	MI/R rat	The interactions of Atg14L-Beclin1-Vps34 complex↓, autophagy↓	[192]
Frulic acid	MI/R rat	The activities of the antioxidant enzymes, ROS↓	[194]	MI rat H9c2 cardiomyocytes	Autophagy↑	[195]
Schizandrol A	AMI mice	Regulate the pathways of glycine, serine and threonine metabolism, lysine biosynthesis, pyrimidine metabolism, arginine and proline metabolism, cysteine and methionine metabolism, valine, leucine and isoleucine biosynthesis	[216]	AD differentiated SH-SY5Y cells or primary hippocampal neurons	PI3K/AKT/mTOR mediated autophagy↓	[218]
Pioglitazone	MI/R mice	Inflammatory changes in adipose tissue↑	[229]	Patients undergoing cardiac surgery for coronary artery bypass grafting and/or valve surgery Human CPCs	Autophagy↓	[232]
Perhexiline	Langendorff-perfused rat	Fatty acid↓, lactate utilization↑	[225]	Immortalized mouse embryo fibroblasts	mTOR mediated autophagy↑	[225]
Dichloroacetate	MI/R rat	Glucose oxidation↑, fatty acid oxidation↓	[226]	MI/R rat	Beclin-1 expression↓, autophagy↓	[90]
Carvedilol	MI/R mice	Glucose uptake and glucose oxidation↑, fatty acid oxidation↓	[227]	AMI rat	Autophagy↑	[227]
Acai	MI/R rat	Alter the substrate selection for mitochondrial oxidation in reperfusion from glucose to fatty acids, maintaining energy metabolism closer to a physiological situation	[249]	Açaí-fed rats	Autophagy↑	[221]
Trimetazidine	MI/R rat	Shift energy production from fatty acid oxidation to glucose oxidation	[219]	MI/R rat	AKT/mTOR-mediated excessive autophagy↓	[219]
Sacubitril/valsartan	Heart failure mice	Energy metabolism-fatty acid metabolism, lipid metabolism, glucose metabolism, and amino acid metabolism	[228]	Hyperthyroidism-induced cardiac hypertrophy rat	Autophagic pathway regulator miR-377↑, autophagy↑	[224]
Nicorandil	MI rat	Lactate accumulation↓	[229]	MI mice	Autophagy↑	[231]
Pioglitazone	MI rat	Lactate accumulation↓	[229]	Renal H/R Normal rat kidney proximal tubular cells NRK-52E	AMPK-mTOR mediated autophagy↑	[232]
Levosimendan	MI/R guinea pig	Lactate↓	[230]	MI/R rat cardiac H9c2 myoblasts	Autophagy↑	[233]
Baicalein	MI/R rat	Improve the energy disorder via increasing the activities of Na^+^-K^+^-ATPase and Ca^++^-ATPase	[234]	Cardiac hypertrophy was induced in mice by injection of ISO for 15 days	Mitophagy receptor FUN14 domain containing 1(FUNDC1) ↑, autophagy↑	[237]
Salidroside	MI/R rat	Regulate energy metabolism	[236]	MI/R rat	Autophagy↓	[239]
Shenmai injection	MI/R primary cardiomyocytes from SD rats	key enzymes and ATP content in the energy metabolism process↑		DOX-induced cardiotoxicity	JNK mediated autophagy formation↓	
Crocetin	MI/R rat	ATPase activities associated with energy metabolism↑	[250]	Human Hepatocyte LO2 Cell	Autophagy↑	[238]
Ferulic acid	MI/R rat	ATP↑	[240]	MI rat H9c2 cardiomyocytes	Autophagy↑	[195]
Ginsenoside Rg1	MI/R rat	ATP↑	[244]	MI/R H9c2	PI3K/AKT/mTOR mediated autophagy↑	[241]
Uridine 5′-triphosphate	MI rat	ATP↑	[242]	MI/R mCherry-LC3 transgenic mice	Autophagy↑	[245]
Hongjingtian Injection	H_2_O_2_-induced H9c2 injury	ATP↑	[41]	MI/R C57BL/6 mice	ROS-mediated autophagic flux↓	[41]
Vitexin	H/R -induced H9c2 injury	ATP↑	[198]	MI/R rat	Autophagy↓	[246]
Puerarin	MI/R H9C2 cells	ATP↑	[193]	H/R-induced H9c2 injury	ANRIL↑, autophagy↓	[205]
Melatonin	H/R H9c2 cells	ATP↑	[247]	MI/R rat	Excessive mitophagy and autophagy↓	[155]
Levosimendan	MI/R guinea pig	ATP↑	[230]	MI/R rat cardiac H9c2 myoblasts	Autophagy↑	[233]

#### 6.3.2 Effects of regulators of amino acid metabolism, glucose metabolism, lipid metabolism on autophagy

In the myocardial injury model, some drugs reduced myocardial injury by regulating amino acid metabolism, lipid metabolism and glucose metabolism, and caused changes in myocardial autophagic flux. For example, schizandrol A and oridonin played a protective role in myocardial injury model by regulating amino acid metabolism and autophagic flux. Specifically, oridonin regulated various metabolic pathways, including glycolysis and BCAA metabolism, and plays a significant role in cardioprotection by regulating energy and amino acid metabolism [214]. Oridonin promoted p21-related autophagic lysosomal degradation, thereby reducing oxidative damage and cardiac hypertrophy [215]. Schizandrol A (SchA) regulated the pathways of glycine, serine, and threonine metabolism; lysine biosynthesis; pyrimidine metabolism; arginine and proline metabolism; cysteine and methionine metabolism; valine, leucine, and isoleucine biosynthesis in AMI [216, 217]. Moreover, SchA attenuated A beta (1-42)-induced autophagy through activating PI3K/AKT/mTOR signaling pathway in AD differentiated SH-SY5Y cells or primary hippocampal neurons [218]. SchA may also inhibit autophagy in cardiomyocytes.

Some metabolic modulators reduce myocardial damage by shifting energy production from fatty acids to glucose oxidation, a process that regulates autophagic flux. For example, TMZ is a promising new generation metabolic agent that works by shifting energy production from fatty acid to glucose oxidation, thereby decreasing oxidative damage. TMZ prevented MI/R injury by inhibiting excessive autophagy via the AKT/mTOR pathway in both in vivo and in vitro model [219]. Euterpe oleracea mart (Acai), a fruit with high antioxidant capacity, also changed the substrate selection for mitochondrial oxidation from glucose to fatty acids to normalize MI/R metabolism by altering energy metabolism during reperfusion [220]. Related studies showed that autophagy markers such as p62, phospho-mTOR, beclin1 and MAP1B-LC3 were mostly up-regulated in acai fed rats. Therefore, we hypothesize that autophagy may also play a role in cardiac autophagy.[221]. Perhexiline, dichloroacetate, carvedilol and sacubitril/valsartan (an angiotensin receptor inhibitor) can reduce myocardial injury [222-224] by shifting energy production from fatty acid to glucose oxidation [90, 225-228] and regulating myocardial autophagic flux. In addition, nicorandil, pioglitazone and levosimendan reduce myocardial injury by inhibiting lactate accumulation [229, 230] and inducing autophagy activation in MI/R model [229, 231-233].

Some drugs have been demonstrated to regulate myocardial autophagic flux and reduce myocardial damage by correcting energy metabolism disorders. Pretreatment with baicalein, shenmai injection (SM), crocetin, atorvastatin and salidroside in MI/R or MI injury models may upregulate key enzymes and restore the depleted ATP content in the energy metabolism process [234-236], causing changes in myocardial autophagic flux, which ultimately leads to reduced myocardial damage [212, 237-239]. For example, baicalein improves the energy disorders by increasing the activity of Na^+^-K^+^-ATPase and Ca^2+^-ATPase in MI/R rats [234]. In mice with cardiac hypertrophy induced by isoproterenol injection for 15 days, baicalein upregulated the mitophagy receptor FUN14 domain containing 1 (FUNDC1) and upregulated autophagy [237]. Salidroside regulated energy metabolism, inhibited myocardial autophagy, and reduced myocardial injury in MI/R [236, 239]. retreatment of MI/R primary cardiomyocytes from SD rats with shenmai injection (SM) may upregulate key enzymes and restore depleted ATP content during the energy metabolism process.[236]. Another study revealed that SM reduces DOX-induced cardiotoxicity by blocking the DOX-induced apoptotic pathway and autophagy [212]. In addition, ferulic acid, ginsenoside Rg1 (Rg1), uridine 5′-triphosphate (UTP), hongjingtian injection (HJT), vitexin, puerarin, melatonin, levosimendan and etomoxir can reduce ATP levels in MI/R rats or H/R H9c2 cells [158, 198, 230, 240-244], and autophagy is regulated to reduce myocardial injury [158, 195, 233, 245-247].

## 7. Future Directions and Conclusions

### 7.1 Current gaps in knowledge and areas for further research

**Figure 5.**
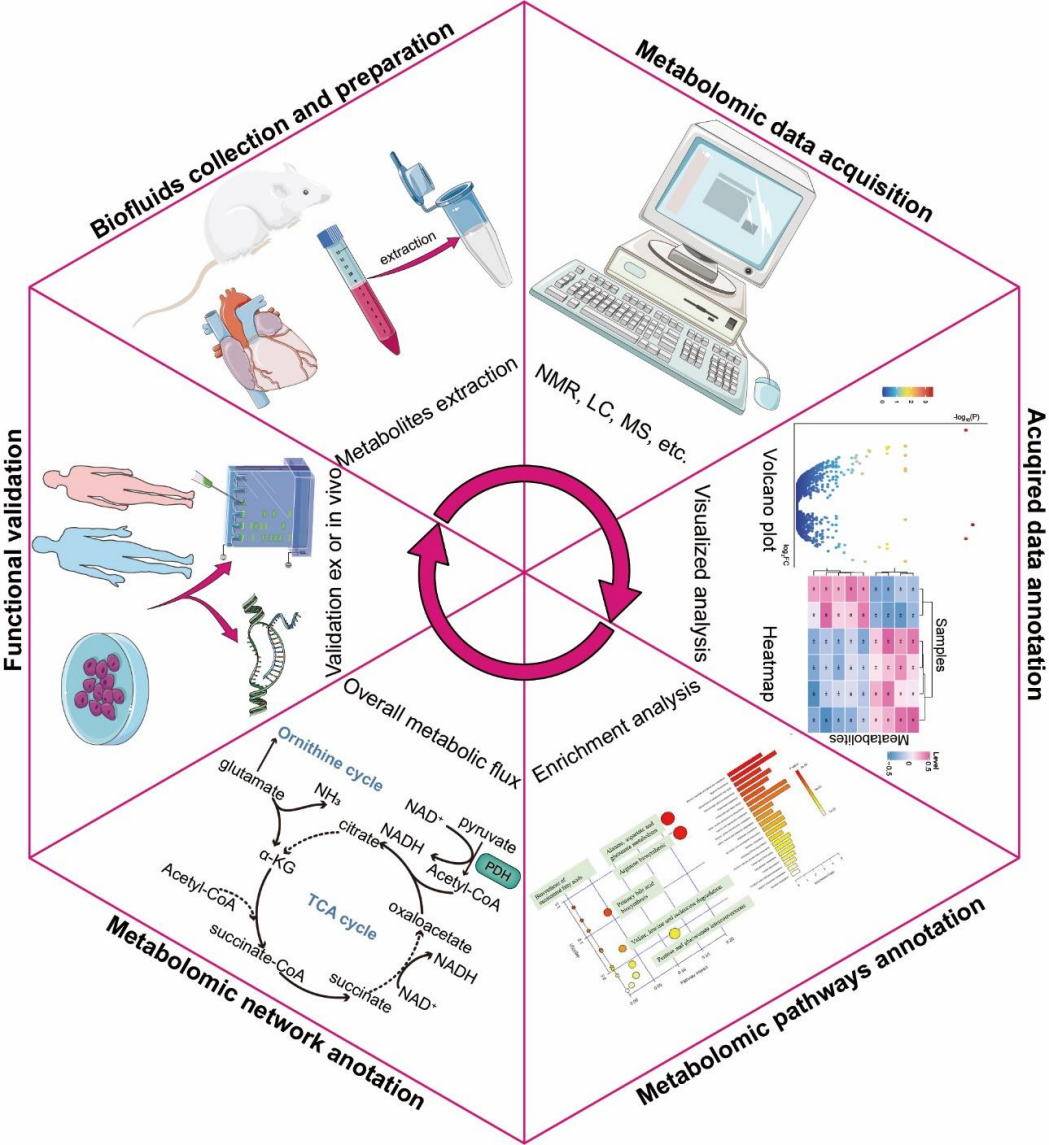
**Workflow of metabolomics**. Metabolomics analysis included biofluid collection and preparation, metabolomic data acquisition, acquired data annotation, metabolomic pathway annotation, metabolomic network annotation, and functional validation.

Metabolomics, as an advanced omics tool, has allowed better understanding of metabolic disturbances in patients globally and broadened our knowledge of related molecules involved in the myocardial ischemia and/or reperfusion stage. Further research is required to identify the autophagy biomarkers using metabolomics. This potent tool would accelerate the discovery of specific biomarkers related to myocardial ischemia and/or reperfusion in clinical practice, which has brought novel options for early diagnosis (Fig. 5). Despite successes in recent years, the metabolomic workflow remains unexplored.

i. In clinical practice, biofluids (such as blood and urine) are typically used for metabolomic analyses. Benefiting from sample availability, biofluid-based metabolomics has greatly aided in the diagnosis and prognostic assessment of patients with MI/R. However, many patients with CVD have associated comorbidities, such as obesity and type 2 diabetes, which may affect their metabolic profile. Under these conditions, biofluid-based metabolic fingerprints may not be able to accurately characterize reperfused hearts. In addition, individual metabolic profiles also reflect dietary habits, drug intake, and other confounding factors, which might contribute to the complexity and inconvenience experienced by doctors. Cardiac arterial blood may provide more accurate samples for metabolomics studies. However, the approach is invasive, Population-based metabolomics might adjust for these potential confounding factors in statistics, enhancing data quality.
ii. In recent decades, many scientists have attempted to integrate molecular datasets, which have greatly helped researchers. However, certain limitations remain. For instance, multiple pivotal pieces of information are unavailable in these public datasets. The choice of detection and analytical platforms depends on the different samples and targets of investigation, instrument availability, and other factors that directly and significantly influence the quality or quantity of metabolite identification and annotation. Therefore, the availability of operational protocols is crucial for further developments. Furthermore, high-density data lead to challenges in data post-processing software, hardware, and data retrieval. Hence, the development of computing techniques to quickly process and integrate different molecular datasets is crucial.

### 7.2 Promising therapeutic approaches for improving myocardial ischemia and reperfusion outcomes

Autophagy disruption plays a pivotal role in various diseases. Therefore, multiple autophagy therapeutics have attracted considerable attention. In MI/R, the maintenance of autophagic flux is critical for attenuating reperfusion injury and reducing myocardial death by ROS elimination. Various assays have been developed to observe and study autophagy and considerable success has been achieved in preclinical studies. However, quantifying autophagic flux remains challenging, especially in clinical practice. Current quantitative approaches are highly dependent on tissues and cells isolated from experimental subjects. MI/R is both invasive and harmful. However, these assays (ex vivo) still face challenges of high sensitivity and throughput. In addition, we highlighted an existing paradox in this review: researchers should focus on the completion of cargo degradation by autolysosomes. However, current detection approaches may be deceiving and cause challenges for scientists. Thus, there is an urgent need for accurate and precise systems to assess autophagic flux in vitro and in vivo. Direct imaging technology and credible biomarkers of autophagy may be reliable solutions for clinical development. Thus, doctors can non-invasively and easily detect and evaluate autophagic flux in patients.

Current research has provided evidence of the cardioprotective effects of metabolic and autophagic modulators. In this review, we systematically elucidated the close coordination between blocked autophagic flux induced by ROS and cardiac metabolism during myocardial ischemia and/or reperfusion. Briefly, drugs targeting autophagic flux can adjust myocardial metabolism, and metabolic modulators affect cardiac autophagy. Considering that there are few effective approaches for detecting autophagic flux in the clinic, assessing autophagic levels by characterizing the metabolic profiles of patients provides a reliable option.

### 7.3 Overall summary and implications for future studies

Taken together, these results indicate that autophagy is regulated by various pathways during ischemia and reperfusion. However, the development of an appropriate assay to assess autophagy in patients remains challenging. Here, we have highlighted the coordination of autophagy and cardiac metabolism, which can help scientists further elucidate the underlying molecular mechanisms and guide future efforts. In our view, using metabolomics to identify autophagy biomarkers can greatly help doctors decide on treatment plans. Moreover, the molecular targeting of autophagy might be a promising cardioprotective strategy in the future, which requires further investigation.
